# Assignment strategies modulate students’ academic performance in an online learning environment during the first and second COVID-19 related school closures

**DOI:** 10.1371/journal.pone.0284868

**Published:** 2023-05-03

**Authors:** Markus Wolfgang Hermann Spitzer, Korbinian Moeller, Sebastian Musslick

**Affiliations:** 1 Department of Psychology, University of Basel, Basel, Switzerland; 2 Centre for Mathematical Cognition, School of Science, Loughborough University, Loughborough, United Kingdom; 3 Leibniz-Institut fuer Wissensmedien, Tübingen, Germany; 4 LEAD Graduate School and Research Network, University of Tuebingen, Tübingen, Germany; 5 Department of Cognitive, Linguistic, and Psychological Sciences, Brown University, Providence, RI, United States of America; 6 Carney Institute for Brain Science, Brown University, Providence, RI, United States of America; 7 Institute of Cognitive Science, Osnabrück University, Osnabrück, Germany; Ahvaz Jundishapur University: Ahvaz Jondishapour University of Medical Sciences, ISLAMIC REPUBLIC OF IRAN

## Abstract

A growing number of studies seek to evaluate the impact of school closures during the ongoing COVID-19 pandemic. While most studies reported severe learning losses in students, some studies found positive effects of school closures on academic performance. However, it is still unclear which factors contribute to the differential effects observed in these studies. In this article, we examine the impact of assignment strategies for problem sets on the academic performance of students (n ≈ 16,000 from grades 4–10 who calculated ≈ 170,000 problem sets) in an online learning environment for mathematics, during the first and second period of pandemic-related school closures in Germany. We observed that, if teachers repeatedly assigned single problem sets (i.e., a small chunk of on average eight mathematical problems) to their class, students’ performance increased significantly during both periods of school closures compared to the same periods in the previous year (without school closures). In contrast, our analyses also indicated that, if teachers assigned bundles of problem sets (i.e., large chunks) or when students self-selected problem sets, students’ performance did not increase significantly. Moreover, students’ performance was generally higher when single problem sets were assigned, compared to the other two assignment types. Taken together, our results imply that teachers’ way of assigning problem sets in online learning environments can have a positive effect on students’ performance in mathematics.

## Introduction

Starting from March 2020, the COVID-19 pandemic led to school closures around the world [1]. These closures required teachers to adopt different approaches to *home schooling* and *distance learning*—with varying degrees of success [2,3]. One such approach are online learning environments, the use of which grew rapidly since the outbreak of the ongoing COVID-19 pandemic [4–8]. For instance, the use of the curriculum-based online learning environment for mathematics *Bettermarks* increased exponentially in Germany among students (age range:10–16) from the point of first school closures [6]. The flexibility provided by such online learning environments, which can be used both remotely as well as in class, affords different strategies of assigning problem sets to students to work on. For instance, in *Bettermarks*, teachers can assign small chunks of problem sets (so called *single problem sets*) within a book (e.g., *Basics of Fractions*) or they can assign large chunks which include the entire book—including all single problem sets of this book—to their students. In addition, students may select problem sets themselves. These different problem set assignment strategies may have modulated students’ performance during school closure. If so, evaluating potential differential effects of assignment strategies on students’ academic performance (e.g., better performance for smaller bits for learning) would help to better understand what makes online learning more successful. To examine this hypothesis, we assessed the effect of different assignment strategies on student’s performance in *Bettermarks* during times of first and second school closures. In the following, we will first elaborate on the impact of school closures on students’ academic achievements before we will outline the details of the current study.

### The effect of school closures on students’ academic performance

A growing number of studies investigated the influence of school closures on students’ academic performance [4–7,9–12]. Most of them reported detrimental effects on academic achievement [9–11], as well as student’s physical [13,14], mental [15–18], and social wellbeing [19–22]. For instance, Engzell et al. (2021) assessed student’s performance in national monitoring examinations [23] before and after school closures in the Netherlands and reported a 60% learning loss based on data from 350,000 students (age range 8–11). The detrimental performance effects observed in this study comport with earlier reports on learning losses during summer holidays [24–27]. In addition, complementing results from Engzell et al. (2021), Schult et al., 2021 reported learning losses for fifth grade students in Germany, equivalent to about one entire month of education after the first closure of schools which lasted for less than three months. Similar effects of school closures were also reported by Maldonado and De Witte (2020), who evaluated the effect of the closure of Flemish schools in Belgium in more than 4,000 students. In line with the two studies mentioned earlier [9,10], their findings revealed significant learning losses in mathematics, specifically for students from low socio-economic backgrounds.

Contrary to the detrimental effects described above, a separate line of studies reported no or even positive effects of school closures on academic performance [4–7,12]. For instance, Gore et al. (2020) found no significant influence of school closures on students’ academic learning outcomes in mathematics in an Australian cohort which included over 4,800 primary school students from New South Wales. In addition, Tomasik et al. (2020) analyzed data from 28,000 students who used an online learning environment before, during, and after school closures in Switzerland. Their results suggested severe learning losses in mathematics—in particular for low-performing Swiss students. However, Tomasik et al. (2020) also observed that some students seemed unaffected by school closures.

Finally, a growing number of studies reported the use of digital technologies to learn from distance during school closures [28–31] and some studies indicated that academic performance in online learning environments improved during school closures [4–6]. For instance, Meeter (2021) examined data from almost 100,000 students from the Netherlands and reported significant learning gains for the period of school closures in an online learning environment for mathematics. Similarly, Van der Velde (2021) investigated students’ performance when learning French in an online environment and found significant learning gains across more than 130,000 students from the Netherlands.

Such performance improvements in online learning have been attributed to a remarkable increase in their usage during school closures and concomitant increase in study time [4,5]. However, a recent within-student analysis of over 2,500 students in Germany (grades 4 to 10; age range:10–16) reported improvements in academic performance while controlling for the extend of online learning and problem set difficulty [6]. Results from this analysis suggest that students performed mathematical problem sets more accurately during the first period of school closures in Germany compared to the same time period in the year before (not affected by the COVID pandemic). Moreover, results from this study suggested a narrowing performance gap, with low-performing students showing more pronounced improvements in performance than already high-performing students [6].

While the above studies substantiate the relevance of online learning environments during school closures, they offer little insight into which factors contributed to the reported learning gains. Some suggested that such positive effects may result from a higher focus when learning at home compared to the classroom which may be nosier and thus more distracting [4,5]. Others argued for the beneficial effects of software integrated features, such as rapid feedback [32–37] or computer-based scaffolding in STEM subjects (i.e., science, technology, engineering, and mathematics) [38–42], both of which may aid learning over and above traditional teaching materials.

Another feature of online learning environments is the flexibility with which they can be used. Teachers may assign problem sets either one by one (i.e., in small chunks) following the progression on the content taught, in bulk (i.e., in larger chunks), or students may even select problem sets themselves. The most common form of assignment in, for instance, *Bettermarks*, are one by one assignments by teachers (65%), followed by bulk assignments by teachers (24%), and self-selected assignments (11%). As teacher-student interaction is known to affect students’ learning outcomes [43], we aimed at evaluating the role of problem set assignment strategies within online learning environments during school closures. In particular, we contrasted the most common form of assignment (single problem sets assigned by teachers) against the two alternative assignment strategies (teachers assign problem sets in bulk or students self-select their problem set) in determining students’ performance outcomes in *Bettermarks*.

### The present study

In this study, we investigated performance-dependent changes as a function of assignment policy by contrasting the most common assignment policy (teachers assigning single problem sets one by one to their students) against two other assignment policies: (1) teachers assigning all problem sets in an online math book at once (e.g., *Basics of Fractions*) or (2) students’ self-selected problem sets. We also examined whether time window-dependent performance changes (i.e., performance before vs. performance during school closures) were affected by the assignment policy. Finally, to evaluate the validity of our results we replicated these analyses for the second period of school closure in Germany and its respective time window in the previous year. As such, this study not only evaluated evidence for the first period of school-closures in Germany but also replicated the analyses for the second school-closure, which reaffirms reported effects. Similar to previous studies on the influence of school closures on learning losses within online learning environments [4,5,7], we compared performance-dependent changes as a function of assignment policy with a between-student analysis approach for the first period of school closures and replicated this analysis for the second period of school closures to examine the robustness of the first results. In addition, this also allowed us to examine whether results for the second period of school closures would be similar to the first period of school closures and thus would generalize the findings to more than one period of school closures. These analyses encompass data from more than 16,000 students (more than 1,900 classes) who calculated on more than 170,000 problem sets.

Based on the set of findings described above [4–6], we expected performance increases due to school closures in the online learning environment. Our hypotheses about the effects of assignment strategy derive from prior studies on spaced and massed learning [44–49]. In this context, it was repeatedly observed that spaced learning—breaking down learning content into smaller bits covered in several sessions (separated by up to several days)—led to significantly better learning outcomes than massed learning reflecting long but fewer learning session on one and the same topic. Against this background one might assume that students’ learning outcomes should be better when teachers’ assign problem sets one-by-one and not all at once. Thus, we expected that assignments of single problem sets, compared to assignments of entire books, may foster greater improvements in performance.

Finally, we were interested in whether assigning single problem sets (the most common assignment policy) or self-selecting single problem sets may have had different effects on performance because these two assignment policies might imply differences in extrinsic vs. intrinsic motivation to complete the respective problem sets [50,51]. In particular, allowing students freedom to choose has been associated with increased intrinsic motivation, effort, and task performance [52–55] with higher intrinsic motivation leading to increased performance [56,57]. Thus, one might expect better performance for students who selected single problem sets themselves as compared to being assigned single problem sets by teachers.

In summary, investigating differences between assignment policies on a large scale, may have important implications on how students best learn mathematics in online learning environments. Thus, we sought to explore whether differences between assignment policies which imply influences of spaced vs. massed learning (i.e., assigning single problem sets vs. all at once) and differences in motivational aspects (i.e., teacher-assigned vs. self-selected problem sets) exist and modulated effects of school closures.

## Methods

### Online learning environment

**Fig 1:**
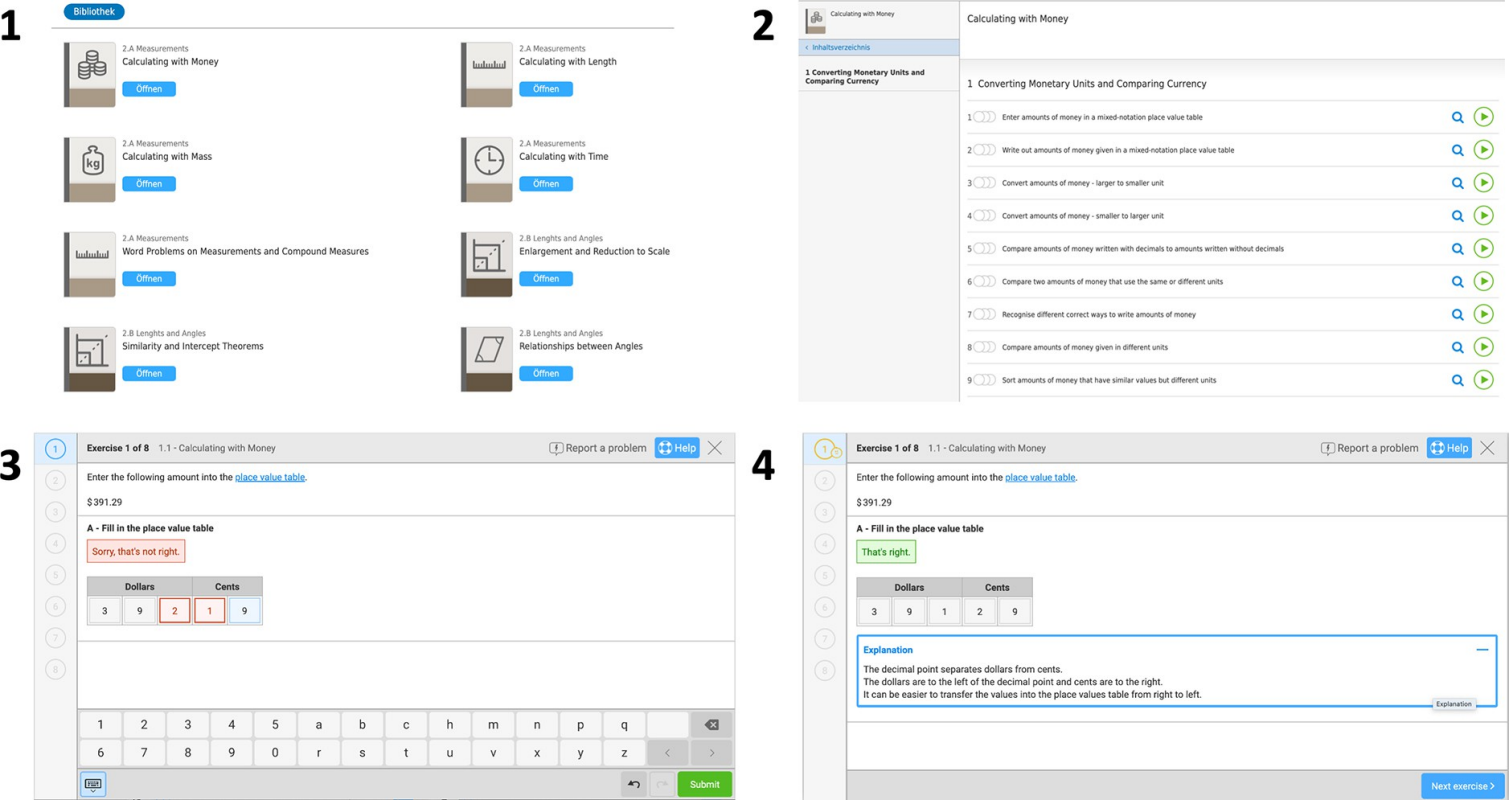
The user interface in Bettermarks. (1) Students and teachers can choose mathematical topics, such as “Calculating with Money” from a library. (2) Each mathematical topic contains several problem sets which contain several individual problems. (3) Immediate feedback is given on every problem. (4) Explanations can be provided.

We made use of data collected with the *Bettermarks* online learning environment. The user interface of *Bettermarks* is depicted in Fig 1. Teachers and students use this software in both private and public schools in all states in Germany, including different school types (i.e., from vocational to academic track schools). The online learning environment contains over 100 different text books covering the curriculum for mathematics in Germany for grades 4 to 10. These virtual mathematics text books cover over 2 000 different mathematical problem sets. Teachers and students with access to the software can freely decide when and how many problem sets they want to work on. *Bettermarks* is used to solve mathematical problem sets online, which can be done inside, but also outside the classroom (e.g., at home). Typically, *Bettermarks* is used to work on problem sets. Two different assignment possibilities exist. Teachers can assign mathematical problem sets (each including eight mathematical problems on average) to their students using the online learning environment. However, students may also work on problem sets on their own, independently of assignments they receive from their teachers. Students receive immediate feedback on each computed problem, indicating whether their answer was correct or not. After completing a problem set, they may choose to repeat it. However, on each new repetition, the parameterization of the problem set changes, and thus, students do not benefit from memorizing the answer of the same problem set.

Students may also leave the system anytime during their learning process. The dataset is entirely anonymous, and thus, it is not possible to identify any personal information from students or teachers (e.g., gender or age). When signing up with the software, each software user agrees that their data will be stored anonymously and used for data analyses. This analysis considered a retrospective assessment of data collected by Bettermarks. All procedures were in accordance with the ethical standards of the national research committee and with the 1964 Helsinki declaration and its later amendments or comparable ethical standards.

**Fig 2:**
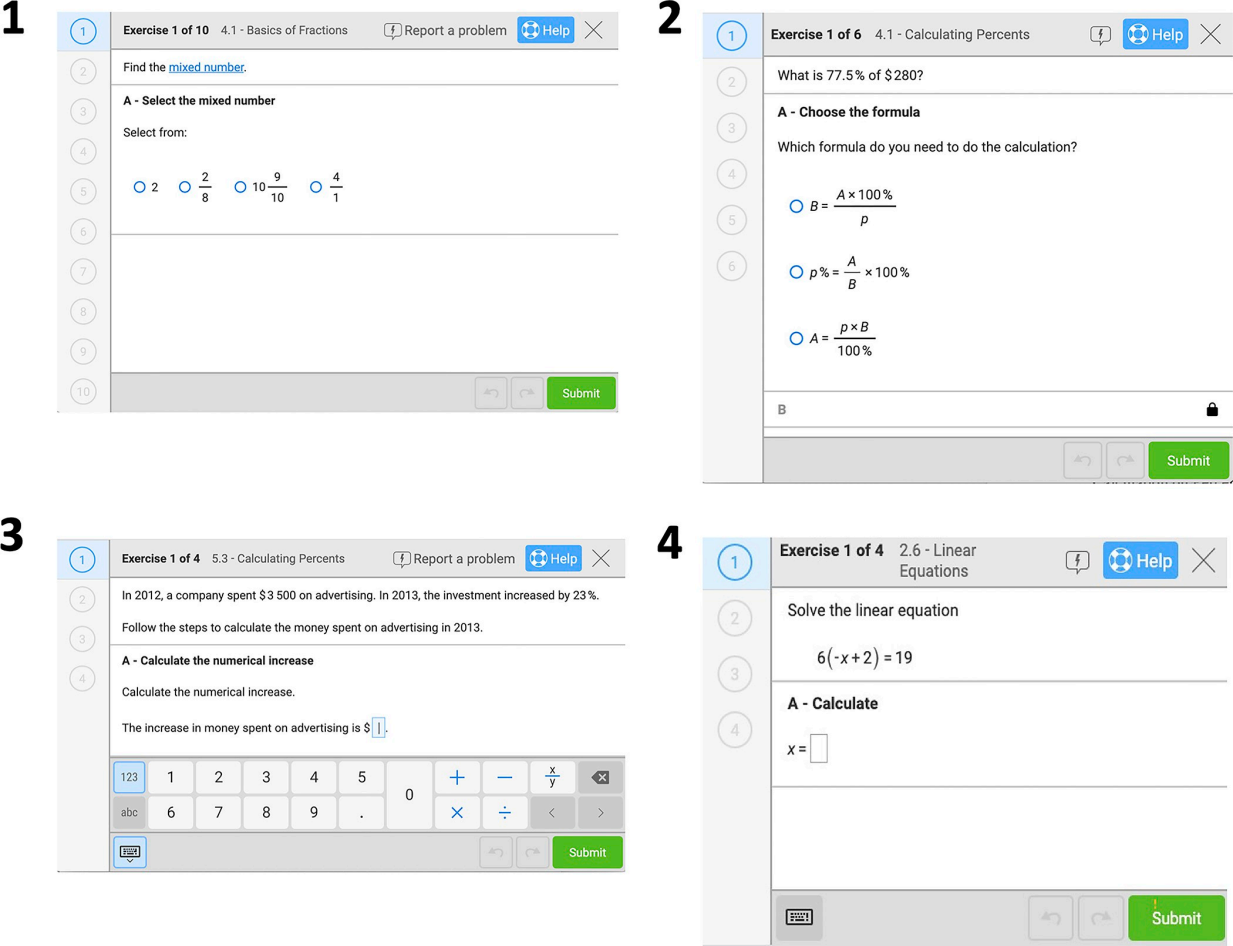
Example problem sets of *Bettermarks*. Example problem sets of the books (A) *Basics of Fractions*, (B, C) *Calculating Percents*, and (D) “*Linear Equations*”.

**Fig 3:**
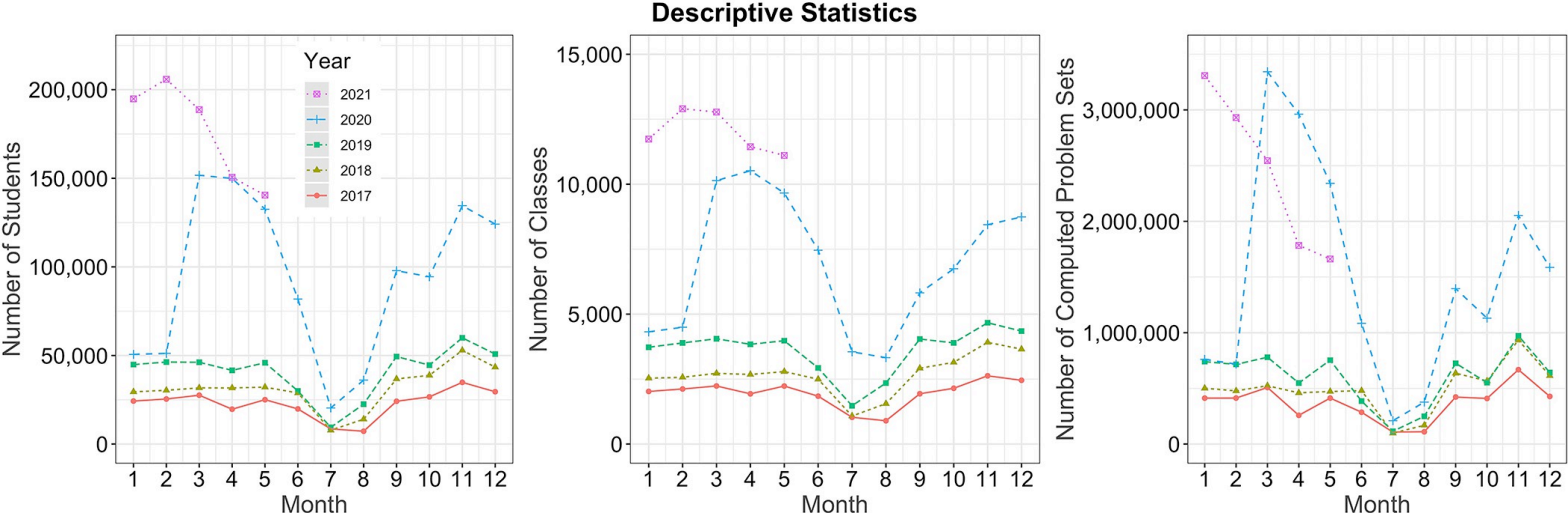
*Bettermarks* usage in Germany over the past five years. Each dot resembles the total number of students (left panel) and classes (middle panel) using the online learning environment, as well as the total number of problem sets completed (right panel) per month of the year. Different colors, lines and dot shape correspond to different years of usage. Note a stark increase in software usage in March 2020 when schools closed for the first time due to the COVID-19 pandemic.

The software covers the curricula of mathematics from classes 4–10 in Germany. Mathematical topics are placed in different books such as *Basics of Fractions*. These books are described in more detail in the S1 Text. The software collects data on each computed problem set on (1) the accuracy of a student; (2) which problem set he/she worked on; (3) whether the problem set was assigned or self-selected; (4) the date the problem set was worked on, and (5) whether students completed the problem set or left the assignment without completion. *Bettermarks* implements an internal incentive structure according to which students gain a star within the system if they complete a problem set with 100% accuracy. They receive a coin if they perform a problem set with more than 60% accuracy. Fig 2 illustrates some example problem sets of the software. Fig 3 depicts its usage over the past five years.

Importantly, the online learning environment did not change over the past years. This allowed us to investigate the performance on problem sets across different time points before and during school closures due to the COVID-19 pandemic.

### Inclusion criteria for sample selection

**Fig 4:**
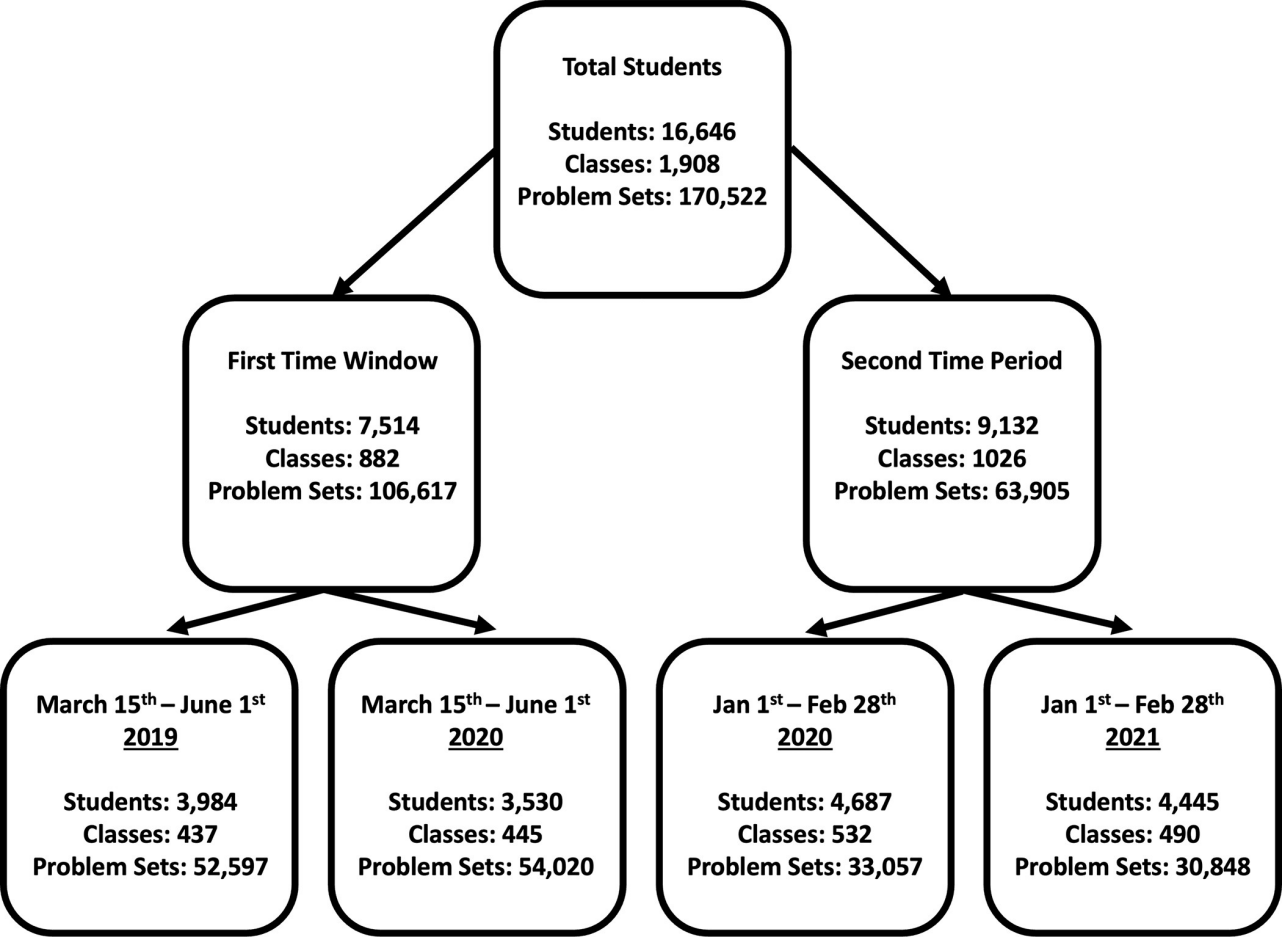
Inclusion criteria for different time periods. Only students who were already registered before first school closures were included in all analyses.

Data considered in the current study (i.e., students, problem sets, dates, and the performance on these problem sets) were selected based on the following criteria which were set prior to data analysis (also see Fig 4). First, we only included students who calculated problem sets in books covering three major mathematical topics, namely fractions, percentages, and linear equations. We predefined these three book topics prior to all analyses and included no other book topics (see S1 Text). Second, we only considered problem sets computed during the first period of school closures in 2020 (March 15^th^, 2020, until June 1^st^, 2020) and the same period in the year before (March 15^th^, 2019, until June 1^st^, 2019). In addition, we included data from the second period of school closures in 2021 (January 1^st^, 2021 until February 28^th^, 2021) and the same time window in the year before (January 1^st^, 2020 until February 28^th^, 2020). Third, we only included students who registered with the software before March 15^th^, 2019. Forth, for each book topic, we only considered students who calculated at least five problem sets of a book, and who worked on a book before school closures *or* during school closures, but not both. In case students repeated a problem set, only the best result of each student on each problem set was included whereas all other repetitions were excluded from the analysis, so to approximate their best performance. Fifth, within a class, the same assignment strategy was always assigned. That is, if students of a class computed problem sets, all of them selected these problem sets on their own or via one of the two assignment types by their teacher. Finally, for single problem set assignments and entire book assignments, we only considered classes with at least 15 students, to assure that we assessed teacher-student interactions within a class context of a minimum size of 15 students. Based on these inclusion criteria, the dataset comprised a total of 16,646 students from 1,908 classes who calculated a total of 170,522 problem sets.

### Independent and dependent variables

Independent variables encompass the categorical variable *time window* which indicated whether schools were closed or not and a categorical *assignment* variable which denoted assignment policy. We additionally controlled for the number of problem sets assigned to students by considering *computed problem sets* as a covariate in our analyses. We compared the most frequently applied assignment policy of teachers assigning single problem sets (labeled as *single problem sets*) with two other possible assignment policies, namely assigning entire books (labeled as *book*) and self-selecting problem sets (labeled as *self-selected problem sets*). Note that teacher-directed assignment policies are independent from the number of students who receive the assignment. In other words, teachers may assign single problem sets or books to either one or multiple students. We considered three different dependent variables that derive from the binary incentive structure of *Bettermarks*. These included (1) a binary *completion* variable indicating whether students completed a problem set or not, irrespective of their performance (2) a binary *stars* variable indicating whether students received a star (i.e., achieved 100% accuracy on a given problem set), and (3) a binary *coins* variable indicating whether students received at least one coin (i.e., achieved at least 60% accuracy on a given problem set).

### Data analysis

We analyzed the dataset using logistic mixed models estimated with the lme4 package [58] within the R software [59,60]. The goal of our analysis was to evaluate whether differences in each dependent variable (completion, stars, or coins) could be observed with respect to the two independent variables time window and assignment, including their interaction (see S1 Text). To account for differences across individuals and classes with respect to the overall effect on each dependent variable, we included a nested random intercept for students and classes. Our mixed effects logistic regression model also included a random intercept for the book name to accommodate differences between books on the same mathematical book topic (e.g., the book topic fractions included the three books: *Basics of Fractions*, *Addition and Subtraction of Fractions*, and *Multiplication and Division of Fractions*), resulting in the general model formula:

dependentvariable∼timewindow*assignmentpolicy+computedproblemsets+(1|class/student)+(1|book).

We applied this model across different analyses, with each analysis contrasting different levels of each independent variable. That is, one set of analyses contrasted the single problem set assignment policy against the book assignment policy, and another set contrasted the single problem set policy against the self-selected assignment policy. We also performed different analyses for different contrasts of time windows: in one set of analyses, we compared the *first* period of school closures in 2020 with the same period a year before and in another set of analyses, we compared the *second* lockdown period in 2021 with the same period in the previous year. This resulted in 3 (three dependent variables) x 2 (assignment policy contrasts) x 2 (time window contrasts) statistical models that we report on below. As six regression analyses were run for the first school closure, we corrected the significance level using Bonferroni correction meaning that we refer to significant results with p-values below .0083. To investigate the robustness of these results, we replicated each analysis for the second period of school closures.

Finally, we conducted post-hoc analyses on the effect of assignment before school closures to investigate whether completion rates, stars and coins gained depended on the assignment strategy (single problem set vs entire book and single problem set vs self-selected problem set). The following model formula with data collected before school closures was considered for these analyses:

dependentvariable∼assignmentpolicy+computedproblemsets+(1|class/student)+(1|book).

## Results

### First shutdown of schools

**Fig 5:**
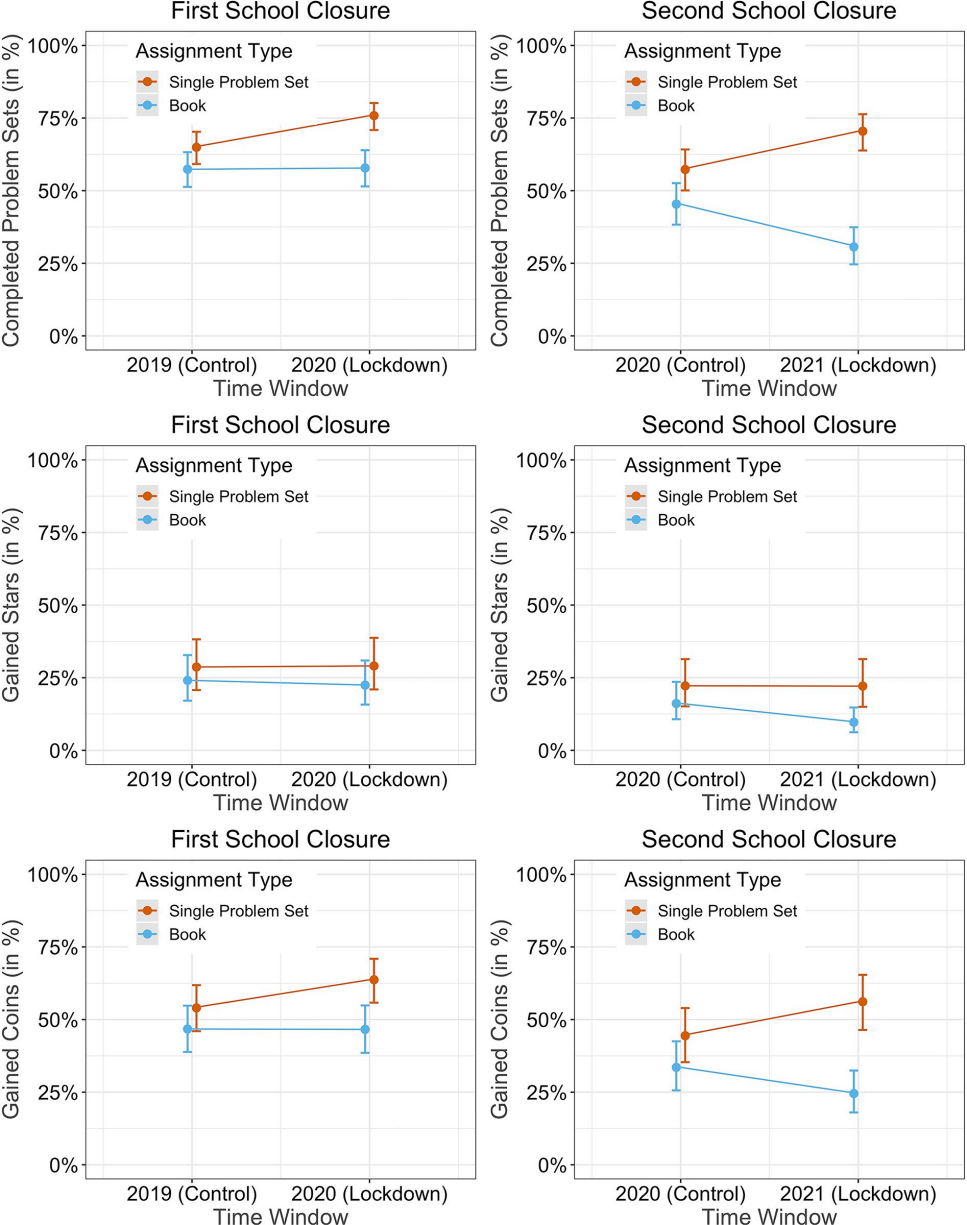
Predicted estimates from the regression models for performance under single problem set assignments (red) and book assignments (lightblue), for the school closure in 2020 (left panels) and 2021 (right panels) compared to the same time periods in the preceding years. Error bars indicate one standard error of the mean (SEM). Students showed greater completion rates, gained more stars, and gained more coins during single problem set assignments, compared to entire book assignments. In addition, students completed more problem sets and gained more coins during the first and second school closure when they were assigned single problem sets compared to the same time period in the previous year. This pattern did not replicate in cases where teachers assigned entire books.

**Fig 6:**
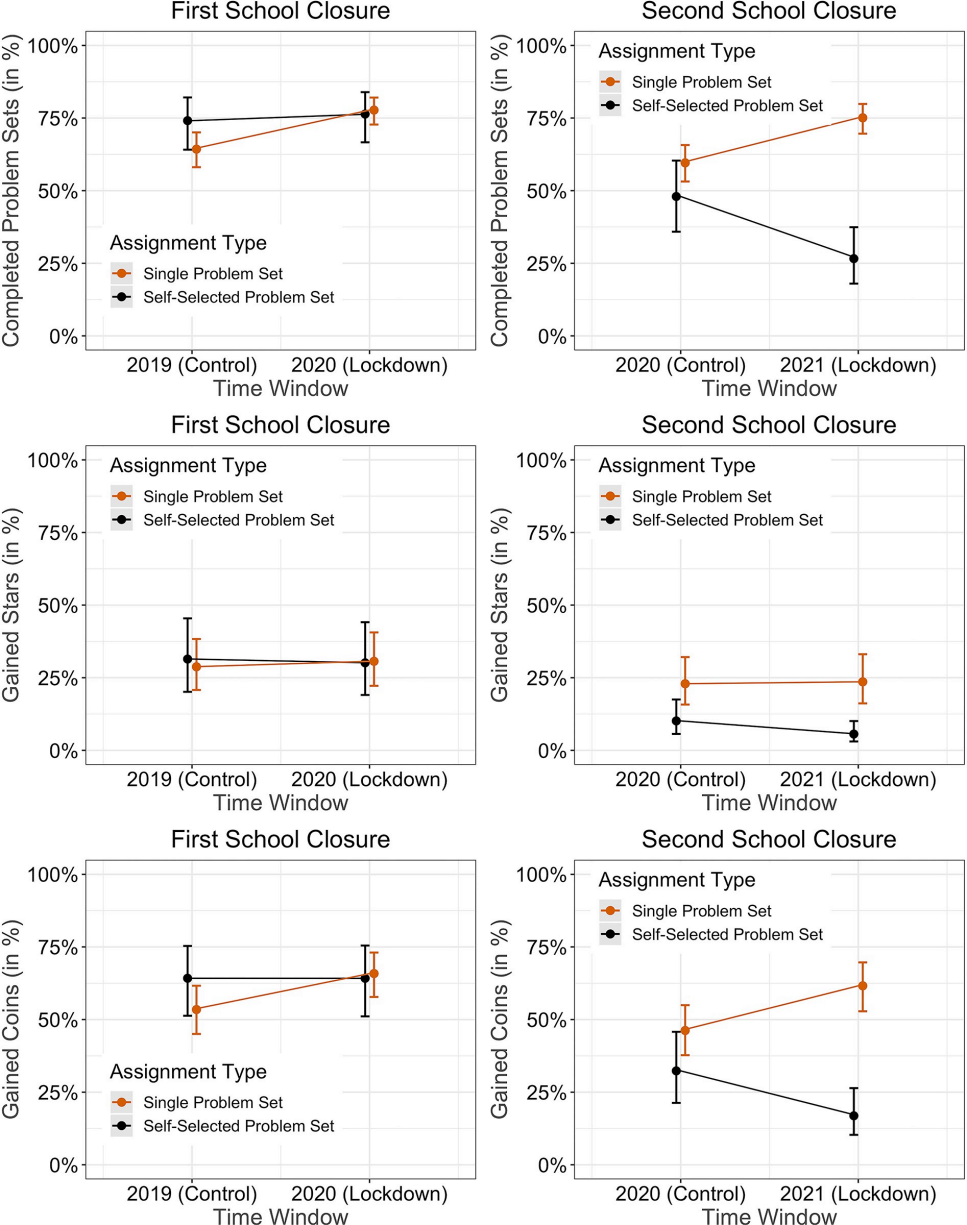
Predicted estimates from the regression models for performance for single problem set assignments (red) and self-selected problem sets (black), for the school closure in 2020 (left panel) and 2021 (right panel) compared to the same time periods in the preceding years. Error bars indicate one SEM. Students completed more problem sets and gained more coins during the first and second school closure if they were assigned single problem sets as compared to the same time periods in the preceding years. We did not observe the same pattern if students self-selected their problem sets.

Results from these analyses are depicted in Figs 5 and 6. All results are also shown in Tables 1–3. In the following sections, we describe the respective analyses.

**Table 1:** Coefficients, estimates (b), confidence intervals, z-values, and p-values for first school closures and second school closures, as well as for single vs. book assignments and single vs. self-selected assignments for completion rates as dependent variable, respectively.

Completion Rate																
	First Closure (single vs. books)				First Closure (single vs. self-selected)				Second Closure (single vs. books)				Second Closure (single vs. self-selected)			
*Coeffcient*	*b*	*Conf*. *Int (95%)*	*z-Value*	*p-Value*	*b*	*Conf*. *Int (95%)*	*z-Value*	*p-Value*	*b*	*Conf*. *Int (95%)*	*z-Value*	*p-Value*	*b*	*Conf*. *Int (95%)*	*z-Value*	*p-Value*
Intercept	0.67	0.40 – 0.94	4.92	**<0.001**	1.14	0.63 – 1.66	4.33	**<0.001**	0.13	-0.18 – 0.44	0.80	0.422	0.16	-0.39 – 0.70	0.56	0.577
Time Window	0.14	0.07 – 0.20	3.94	**<0.001**	0.20	0.13 – 0.26	6.12	**<0.001**	-0.01	-0.09 – 0.06	-0.35	0.730	-0.05	-0.14 – 0.03	-1.31	0.191
Assignment	0.29	0.24 – 0.34	11.63	**<0.001**	-0.10	-0.50 – 0.30	-0.47	0.637	0.54	0.49 – 0.59	22.31	**<0.001**	0.65	0.21 – 1.08	2.93	**0.003**
Computed Problem Sets	-0.01	-0.00– 0.00	-1.49	0.136	-0.00	-0.00 – -0.00	-2.30	**0.022**	-0.00	-0.00 – 0.00	-1.76	0.079	-0.01	-0.00 – 0.00	-1.09	0.274
Time Window:Assignment	0.13	0.08 – 0.18	5.20	**<0.001**	0.14	0.07 – 0.20	4.24	**<0.001**	0.30	0.25 – 0.35	12.48	**<0.001**	0.41	0.33 – 0.49	9.92	**<0.001**

**Table 2:** Coefficients, estimates (b), confidence intervals, z-values, and p-values for first school closures and second school closures, as well as for single vs. book assignments and single vs. self-selected assignments for stars as dependent variable, respectively.

Stars																
	First Closure (single vs. books)				First Closure (single vs. self-selected)				Second Closure (single vs. books)				Second Closure (single vs. self-selected)			
*Coeffcient*	*b*	*Conf*. *Int (95%)*	*z-Value*	*p-Value*	*b*	*Conf*. *Int (95%)*	*z-Value*	*p-Value*	*b*	*Conf*. *Int (95%)*	*z-Value*	*p-Value*	*b*	*Conf*. *Int (95%)*	*z-Value*	*p-Value*
Intercept	-1.09	-1.54 – -0.65	-4.84	**<0.001**	-0.82	-1.46 – -0.18	-2.52	**0.012**	-1.67	-2.16 – -1.19	-6.79	**<0.001**	-1.93	-2.60 – -1.26	-5.65	**<0.001**
Time Window	-0.02	-0.08 – 0.05	-0.55	0.586	0.01	-0.06 – 0.07	0.22	0.827	-0.15	-0.22 – -0.07	-3.81	**<0.001**	-0.15	-0.24 – -0.06	-3.22	**0.001**
Assignment	0.15	0.10 – 0.19	5.94	**<0.001**	-0.03	-0.45 – 0.40	-0.12	0.906	0.34	0.29 – 0.39	13.55	**<0.001**	0.66	0.22 – 1.09	2.94	**0.003**
Computed Problem Sets	0.00	-0.00 – 0.00	0.91	0.364	-0.00	-0.00 – 0.00	-0.27	0.786	0.00	-0.00 – 0.00	1.64	0.101	0.00	-0.00 – 0.00	1.55	0.121
Time Window:Assignment	0.03	-0.02 – 0.08	1.12	0.264	0.04	-0.03 – 0.10	1.14	0.255	0.15	0.10 – 0.19	5.78	**<0.001**	0.17	0.08 – 0.26	3.65	**<0.001**

**Table 3:** Coefficients, estimates (b), confidence intervals, z-values, and p-values for first school closures and second school closures, as well as for single vs. book assignments and single vs. self-selected assignments for coins as dependent variable, respectively.

Coins																
	First Closure (single vs. books)				First Closure (single vs. self-selected)				Second Closure (single vs. books)				Second Closure (single vs. self-selected)			
*Coeffcient*	*b*	*Conf*. *Int (95%)*	*z-Value*	*p-Value*	*b*	*Conf*. *Int (95%)*	*z-Value*	*p-Value*	*b*	*Conf*. *Int (95%)*	*z-Value*	*p-Value*	*b*	*Conf*. *Int (95%)*	*z-Value*	*p-Value*
Intercept	0.14	-0.20 – 0.48	0.78	0.433	0.55	-0.03 – 1.13	1.87	0.061	-0.38	-0.78 – 0.01	-1.90	0.058	-0.49	-1.10 – 0.12	-1.56	0.118
Time Window	0.10	0.03 – 0.17	2.85	**0.004**	0.13	0.07 – 0.19	4.03	**<0.001**	0.01	-0.07 – 0.09	0.19	0.853	-0.06	-0.14 – 0.03	-1.36	0.175
Assignment	0.25	0.20 – 0.30	10.15	**<0.001**	-0.09	-0.52 – 0.33	-0.44	0.663	0.46	0.41 – 0.50	19.07	**<0.001**	0.66	0.21 – 1.12	2.86	**0.004**
Computed Problem Sets	-0.00	-0.00 – 0.00	-0.40	0.688	-0.00	-0.00 – 0.00	-1.04	0.297	-0.00	-0.00 – 0.00	-1.22	0.224	-0.00	-0.00 – 0.00	-0.29	0.774
Time Window:Assignment	0.10	0.05 – 0.15	4.20	**<0.001**	0.13	0.07 – 0.19	4.04	**<0.001**	0.23	0.18 – 0.27	9.52	**<0.001**	0.37	0.29 – 0.45	8.73	**<0.001**

#### Completion rate (single problem sets vs. entire books)

The main effect of time window was significant (*b* = 0.14; *z* = 3.94; *p* < .001), with more completed problem sets during school closure as compared to the same time window in the previous year. The significant main effect of assignment (*b* = 0.29; *z* = 11.62; *p* < .001) reflected higher completion rates if individual problem sets were assigned compared to books assignments. The main effect of computed problem sets was not significant (*b* = -.001; *z* = -1.49; *p* = .136). Finally, the interaction of time window and assignment was significant (*b* = .13; *z* = 5.20; *p* < .001), suggesting that the difference in completion rates between school closures and control periods was larger for students who got assigned single problem sets versus entire books.

We additionally conducted a post-hoc analysis on the effect of assignment strategy for the time period before school closures, controlling for number of assignments. Results of this logistic mixed model revealed a significant effect of assignment (*b* = 0.15; *z* = 5.16; *p* < .001), with higher completion rates on single problem set assignments compared to entire book assignments. The main effect of computed assignments was significant (*b* = -0.01; *z* = -3.00; *p* = .003) with lower completion rates when students computed more assignments.

#### Stars (single problem sets vs. entire books)

The main effect of time window was significant (*b* = -.02; *z* = -0.55; *p* = .586). The main effect of assignment was significant (*b* = .15; *z* = 5.94; *p* < .001), with more stars gained on single problem set assignments compared to book assignments. The main effect of computed problem sets was not significant (*b* < .001; *z* = 0.91; *p* = .364). The interaction of time window and assignment was not significant (*b* = .03; *z* = 1.12; *p* = .264).

As in the previous analysis, we carried out a post-hoc analysis on the effect of assignment before school closures. Results of the logistic mixed model with computed problem sets as covariate revealed a significant effect of assignment (*b* = .12; *z* = 3.83; *p* = .001), with more coins gained before school closures on single problem set assignments compared to book assignments. The main effect of computed problem sets was not significant (*b* < .001; *z* = -0.20; *p* = .844).

#### Coins (single problem sets vs. entire books)

The main effect of time window was significant (*b* = .09; *z* = 2.85; *p* = .0043), with overall more coins gained during school closure as compared to the same time window in the previous year. The main effect of assignment was significant (*b* = .25; *z* = 10.15; *p* < .001), with more coins gained on single problem set assignments compared to book assignments. The main effect of computed problem sets was not significant (*b* < -.001; *z* = -0.40; *p* = .688). The interaction of time window and assignment was significant (*b* = .10; *z* = 4.19; *p* < .001) suggesting that students who got assigned single problem sets showed a greater difference in acquired coins as a function of time window, as compared to students who got assigned entire books.

We conducted a post-hoc analysis to investigate the effect of assignment before school closures in isolation. Results of this logistic regression model revealed a significant effect of assignment (*b* = .14; *z* = 4.68; *p* < .001), with more coins gained before school closures on single problem set assignments compared to book assignments. The main effect of computed problem sets was not significant (*b* = .002; *z* = -1.00; *p* = .050).

#### Completion rate (single problem sets vs. self-selected problem sets)

Again, the main effect of time window was significant (*b* = 0.20; *z* = 6.12; *p* < .001) indicating that overall, students completed more problem sets during school closure as compared to the same time window in the previous year. On the other hand, the main effect of assignment was not significant (*b* = -0.10; *z* = -0.47; *p* = .637). The main effect of computed problem sets was not significant (*b* < .002; *z* = -2.29; *p* = .022). The interaction of time window and assignment was significant (*b* = 14; *z* = 4.24; *p* < .001). The interaction indicates that the difference in completion rate as a function of school closures was larger for students who got assigned single problem sets by their teachers compared to students who selected their problem sets.

The post-hoc analysis on the main effect of assignment before school closures was not significant (*b* = -0.45; *z* = -1.85; *p* = .064). The main effect of computed problem sets was significant (*b* = -0.005; *z* = -2.96; *p* = .003) with lower completion rates when more assignments were computed.

#### Stars (single problem sets vs. self-selected problem sets)

The main effect of time window was not significant (*b* = .007; *z* = 0.22; *p* = .827). The main effect of assignment was not significant (*b* = -.03; *z* = -0.12; *p* = .906). The main effect of computed problem sets was not significant (*b* < -.001; *z* = -0.27; *p* = .786). The interaction of time window and assignment was not significant (*b* = .04; *z* = 1.14; *p* = .255).

The post-hoc analysis on the main effect of assignment before school closures did not reveal a significant main effect for assignment (*b* = -0.19; *z* = -0.82; *p* = .412). The main effect for computed problem sets was also not significant (*b* < -0.01; *z* = -1.10; *p* = .271).

#### Coins (single problem sets vs. self-selected problem sets)

The main effect of time window was significant (*b* = .13; *z* = 4.03; *p* < .001), with overall more coins gained during school closure as compared to the same time window in the previous year. The main effect of assignment was not significant (*b* = -.09; *z* = -0.44; *p* = .662). The main effect of computed problem sets was not significant (*b* < -.001; *z* = -1.04; *p* = .297). The interaction of time window and assignment was significant (*b* = .13; *z* = 4.05; *p* < .001) indicating that students who got assigned single problem sets by their teachers showed a greater difference in acquired coins as a function of time window relative to students who self-selected their problem sets.

The post-hoc analysis on the main effect of assignment before school closures did not reveal a significant main effect for assignment (*b* = -0.43; *z* = -1.77; *p* = .077). The main effect for computed problem sets was also not significant (*b* < -0.01; *z* = -2.11; *p* = .034).

### Second lockdown

Figs 5 and 6 as well as Tables 1–3 show the results of the analyses. Each analysis is described in the following sections.

#### Completion rate (single problem sets vs. entire books)

The main effect of time window was not significant (*b* = -.01; *z* = -.35; *p* = .73). The main effect of assignment was significant (*b* = .54; *z* = 22.31; *p* < .001), with higher completion rates on problem sets if they were assigned one by one as opposed to as entire books. The main effect of computed problem sets was not significant (*b* = -.001; *z* = -1.76; *p* = .079). The interaction of time window and assignment was significant (*b* = .30; *z* = 12.48; *p* < .001), suggesting that the difference in completion rates as a function of time window was larger for single problem set assignments as compared to book assignments.

The post-hoc analysis on the main effect of assignment before school closures was significant (*b* = -0.27; *z* = 5.34; *p* < .001), with higher completion rates on single problem set assignments compared to entire book assignments. The main effect of computed problem sets was not significant (*b* < -0.01; *z* = -0.05; *p* = .960).

#### Stars (single problem sets vs. entire books)

The main effect of time window was significant (*b* = -.15; *z* = -3.81; *p* < .001), with less stars gained during school closure. The main effect of assignment was significant (*b* = .34; *z* = 13.55; *p* < .001), with more stars gained on single problem set assignments, compared to entire books assignments. The main effect of computed problem sets was not significant (*b* < .001; *z* = 1.64; *p* = .101). The interaction of time window and assignment was significant (*b* = .15; *z* = 5.78; *p* < .001), indicating that the difference between stars gained during the two time windows was more expressed for single problem set assignments as compared to book assignments.

The post-hoc analysis on the main effect of assignment before school closures was significant (*b* = 0.21; *z* = 3.74; *p* < .001), with more stars gained on single problem set assignments compared to entire book assignments. The main effect of computed problem sets was not significant (*b* < 0.01; *z* = 0.87; *p* = .375).

#### Coins (single problem sets vs. entire books)

The main effect of time window was not significant (*b* = .01; *z* = .19; *p* = .853). The main effect of assignment was significant (*b* = .46; *z* = 19.07; *p* < .001), with more coins gained on single problem set assignments compared to book assignments. The main effect of computed problem sets was not significant (*b* < -.001; *z* = -1.22; *p* = .224). The interaction of time window and assignment was significant (*b* = .23; *z* = 9.52; *p* < .001) with the difference in coins gained between the two time windows being more pronounced for single problem set assignments as opposed to book assignments.

The post-hoc analysis on the main effect of assignment before school closures was significant (*b* = 0.22; *z* = 4.44; *p* < .001), with more coins gained on single problem set assignments compared to entire book assignments. The main effect of computed problem sets was not significant (*b* < 0.01; *z* = 1.04; *p* = .297).

#### Completion rate (single problem sets vs. self-selected problem sets)

The main effect of time window was not significant (*b* = -.05; *z* = -1.31; *p* = .191). The main effect of assignment was significant (*b* = .65; *z* = 2.93; *p* = .003), with higher completion rates for single problem set assignments as compared to book assignments. The main effect of computed problem sets was not significant (*b* = -.001; *z* = -1.09; *p* = .274). The interaction of time window and assignment was significant (*b* = .41; *z* = 9.92; *p* < .001), suggesting greater differences in completion rate as a function of time window if problem sets were assigned one by one as opposed to being self-selected.

The post-hoc analysis on the main effect of assignment before school closures was not significant (*b* = -0.24; *z* = 0.63; *p* = .526). The main effect of computed problem sets was not significant (*b* < -0.01; *z* = -0.71; *p* = .478).

#### Stars (single problem sets vs. self-selected problem sets)

The main effect of time window was significant (*b* = -.15; *z* = -3.22; *p* = .845), with less stars gained during school closure. The main effect of assignment was significant (*b* = .66; *z* = 2.94; *p* = .003), with more stars gained if single problem set were assigned as compared to being self-selected. The main effect of computed problem sets was not significant (*b* < .001; *z* = 1.55; *p* = .121). The interaction of time window and assignment was significant (*b* = .17; *z* = 3.65; *p* < .001), indicating that the lockdown period was associated with more stars compared to the same time frame in the previous year if problem sets were assigned one by one by teachers.

The post-hoc analysis on the main effect of assignment before school closures was not significant (*b* = -0.15; *z* = 0.39; *p* = .696). The main effect of computed problem sets was not significant (*b* < -0.01; *z* = -0.17; *p* = .865).

#### Coins (single problem sets vs. self-selected problem sets)

The main effect of time window was not significant (*b* = -.06; *z* = 2.71; *p* = .007). The main effect of assignment was significant (*b* = .66; *z* = 2.86; *p* = .004), with more coins gained on single problem set assignments, compared to self-selected problem sets. The main effect of computed problem sets was not significant (*b* < -.001; *z* = -0.29; *p* = .774). The interaction of time window and assignment was significant (*b* = .37; *z* = 8.73; *p* < .001) with the difference in coins gained between the two time windows being more pronounced for single problem set assignments by teachers.

The post-hoc analysis on the main effect of assignment before school closures was not significant (*b* = 0.05; *z* = 0.13; *p* = .894). The main effect of computed problem sets was not significant (*b* < 0.01; *z* = 0.55; *p* = .581).

## Discussion

In this study, we investigated the influence of teachers’ assignment policies during school closures on the performance of students (grade 4–10; age range:10–16) in an online learning environment for mathematics with a between-student analysis approach. We observed that if teachers assigned single problem sets to students, the probability of completing a problem set, as well as the probability of achieving at least 60% accuracy on a given problem set was significantly and consistently *higher* for students who computed these single problem sets during the first (i.e., 15.03. to 1.06.2020) and second period of school closures (i.e., 01.01.to 28.02.2021) in Germany as compared to students who computed the same problem sets during the same period the year before, respectively. However, we did not find this effect if teachers assigned entire books or if students self-selected problem sets. We observed this effect across three mathematical topics (i.e., fractions, percentages and interest, and linear equations; see S1 Text). Taken together, these results indicate that the beneficial effects of assigning single problem sets persist across two different periods of school closures and across different mathematical topics and pertain to students’ completion rates, as well as their performance (achieving at least 60% accuracy).

It is noteworthy that this study differed from previous analyses of student performance in the same learning software [6,61,62]. An important difference pertains to the way in which we operationalized students’ performance. In an earlier study, we measured students’ performance in terms of their average error rate, normalized by problem set difficulty [6]. Here, we employed binary factors coding students’ accuracy (e.g., whether they completed a problem set, gained a star, or gained a coin) as we restricted the analysis to specific mathematical contents which were studied most frequently within the online learning environment. An advantage of such an approach is that we were able to evaluate whether our analyses replicate across book topics. The three mathematical topics (fractions, percentages and interest, and linear equations) considered are of tremendous importance for students to perform well on as they have been observed to be robust predictors for future mathematical achievement [63–67], socio-economic status and overall income during adulthood [68–72].

Another advantage of this analysis is the assessment of completed problem sets in combination with students’ performance on mathematical problem sets. This allowed us to examine whether students completed more and got better on these completed problem sets. Without considering completion rates in online learning environment, it may be that students just complete easy problem sets, but do not complete rather challenging problem sets. Thus, finding that students completed more problem sets *and* increased their performance at the same time on these completed problem sets compared to two previous cohorts suggests that students actually performed better. If students would have shown increased performance but decreased completion rates, then this could have suggested that students just completed rather simpler problem sets.

Finally, we investigated whether the performance of two cohorts of students (before each lockdown) differed from another two cohorts of students (during each lockdown) with respect to completion rates, achieving 60% accuracy and achieving 100% accuracy on the same problem sets. We considered the performance on specific mathematical topics with a between-student analysis approach (akin to previous analyses on COVID-19 related performance changes [4,5,7]) as improvements on the same problem sets within the same students over longer time horizons would have been expected.

The reported analyses may shed light on previously observed performance improvements of students using online learning environments during the first COVID-19 related school closure in 2020 [4–6]. These observations seem unexpected given that most studies suggest detrimental effects of school closures [9–11]. As such, the results of the present study suggest that students may learn better during school closures when small bits of information were assigned.

The beneficial effects of teachers assigning problem sets one after another, as opposed to assigning them in bundles or letting students self-select, highlight the importance of how learning materials should be presented to students. In particular, these results suggest that assigning smaller bits of learning content in terms of smaller sets of problem sets as compared to entire books as well as compared to letting students self-select problem sets was most beneficial for students’ mathematical learning.

As such, our results are in line with previous evidence suggesting that spacing out learning content over time, provided to students in small chunks, leads to enhanced learning outcomes [44–49]. Moreover, our results rather speak in favor of a large effect teacher incentives may have, as single problem set assignments were associated with overall increased performance and increased completion rates compared to self-selected assignments. These results may be explained by motivational theories of effort allocation which propose a positive connection between incentives and academic performance [73–75]. Based on these theories, one may speculate that students’ performance depends on incentives provided by the teachers. More precisely, students may be more extrinsically motivated to complete and perform well on problem sets when they were assigned by their teachers (e.g., as part of homework) compared to when they select to perform these problems themselves. Importantly, this rather extrinsic motivational view does not speak against intrinsic motivational effects which the self-selection of problem sets might have [76], but it may be that the effect of extrinsic incentives was larger than the effect of intrinsic incentives. Nevertheless, the positive effect single problem set assignments had, compared to self-selected problem sets, which increased during school closures, highlights the important role of teachers within online learning environments—especially when teachers and students do not see each other in school.

Importantly, students were exposed to the same learning content (the three different mathematical topics fractions, percentages and interest, and linear equations), independent of the assignment strategy (i.e., single problem sets, books, and self-selected problem sets). Thus, overall students from different assignment strategies should have been exposed to problem sets of the same difficulty. To investigate the robustness of our results across different book topics, we ran further control analyzes replicating our original analyses for each book topic separately (see S1 Text). These results showed that the obtained pattern of results was replicated for each book. Thus, differences in difficulty between books did not influence the effect of assignment strategy. Therefore, we are confident our results are not biased by differences in difficulty between book topics.

Another important avenue for future investigation is the effect of teacher-student interactions within online learning environments on students’ performance. For instance, teacher-student interactions within classrooms have been reported as a key source eliciting mathematical anxiety [77,78] which in turn was reported to reduce mathematical performance [79–82]. In contrast, our results suggest that students who got problem sets assigned by their teachers via an online learning platform outperformed students who studied on their own (i.e., with no teacher-student interaction). Thus, future research may investigate whether teacher-student interactions within online learning environments may elicit less math anxiety such that online teacher-student interactions may reduce negative aspects such as mathematical anxiety.

When interpreting the results of the present study some limitations need to be considered. First, it should be acknowledged that performance improvements observed in this study may also be influenced by other factors such as an increase in the overall usage of learning software during lockdowns and school closures [61,83,84]. However, control analyses indicated that the mere degree of software usage did not explain observed differences between time windows. Aside from software usage, one might argue that students exhibited different degrees of motivation in the different time windows, leading to differences in performance [73–75]. The latter may result from the fact that online exercises were the only exercises available during school closures. Other potentially confounding factors include increased support from family members when studying at home, as well as a higher focus when learning from home [4,5]. Such motivational factors might indeed have driven the main effect of time window. However, even if such motivational factors are at play, one would not necessarily expect these to differentially affect the interaction between time window and problem set assignment. That is, we observed selective performance improvements when single problem sets were assigned. Third, our results stem from students from 10–16 years of age and are thus limited to this specific age range. An interesting future avenue would be to test the generalizability of our results to older or younger cohorts of students who studied with an online learning environment during COVID-19 related school closures.

Another limitation of this study is that the data do not include information on why some teachers assigned single problem sets whereas others assigned problem sets in bundles. The learning environment offers both assignment policies and the data suggest that both policies are used by teachers. Nevertheless, assigning single problem set leads to higher completion rates and higher probabilities of gaining a coin (60% accuracy) or a star (100% accuracy) within the online learning environment. Importantly, this effect was already present before school closures. During both school closures, the positive effect of assigning single problem sets seemed to be increased. The present findings thus point toward a beneficial way of how to assign mathematical problem sets to students from classes 4–10 (age range 10–16) best—in small bits rather than big bundles. These results are in line with previous studies on spaced learning [44–49] which repeatedly showed that assigning smaller bits of learning content into smaller bits led to increased learning outcomes compared to massed learning (few but learning sessions).

Working with data obtained from online learning environments may not always allow to control for specific influencing variables such as socioeconomic status, gender, or age as these may be considered sensitive information and may not be shared. Additionally, these data may typically not incorporate specific aspects such as how teachers incentivize their students as well as additional information on students’ general traits and abilities (e.g., self-regulated learning abilities), which were repeatedly observed to influence students’ performance in general [85], and specifically during school closures [86,87]. For instance, the reported results of differential outcomes depending on the assignment strategy may be explained by self-regulated learning. In particular, the decision-load for bulk assignments as well as when students self-selected problem sets (note that students self-select problem sets from a book which is the same as a bulk of assignments) is much higher, so students may struggle to make the right choice where to begin when learning in a self-regulated way. Conversely, when provided with particular small problem sets, students can more easily motivate themselves to perform them because they don’t have to make as many decisions about what to start with. Nevertheless, analyzing these data does allow to evaluate average trends estimated from student samples which are typically considerably larger than sample sizes from experimental studies and in the present case worked on curricular-based mathematical problem sets. As such, investigating such large-scale data from online learning environments (for other online learning environments see: [5,7,88,89]) comes with a trade-off between not being able to control for covariates typically assessed in experimental research (e.g., age and gender) and investigating several subgroups in the population (e.g., males vs. females or groups of differing socioeconomic background), and the benefit of being able to examine data from thousands of students providing information on general trends in the (student) population (classes 4–10; age-range: 10–16).

## Conclusion

In the present study, we evaluated the influence of assignment policy on students’ (classes 4–19; age-range 10–16) performance in an online learning environment for mathematics during school closures. Results suggest performance improvements during school closures due to the COVID-19 pandemic, relative to the years before. Importantly, however, our results also suggest that the degree of students’ improvements was specified by the way students were assigned problem sets: We observed significant performance benefits if students were assigned problem sets one by one as compared to in entire books or having students self-select problem sets. These results indicated that assigning smaller bits of information seemingly was a beneficial strategy during school closures during the pandemic. The results also highlight the importance of teachers for online learning environments—a finding that encourages further research on the exact role of teacher behavior within online learning environments.

## Supporting information

S1 Text (DOCX)

## References

[1] UNESCO. Education: From disruption to recovery. School clusures caused by Coronavirus (Covid-19). 2020.

[2] Hammerstein S, König C, Dreisörner T, Frey A. Effects of COVID-19-Related School Closures on Student Achievement-A Systematic Review. Front Psychol. 2021;12: 1–8. doi: 10.3389/fpsyg.2021.746289 34603162 PMC8481663

[3] König C, Frey A. The Impact of COVID-19-Related School Closures on Student Achievement—A Meta-Analysis. Educ Meas Issues Pract. 2022;41: 16–22. doi: 10.1111/emip.12495

[4] Velde M van der, Sense F, Spijkers R, Meeter M,… Lockdown Learning: Changes in Online Study Activity and Performance of Dutch Secondary School Students during the COVID-19 Pandemic. 2021. Available: https://psyarxiv.com/fr2v8/.

[5] Meeter M. Primary school mathematics during Covid-19: No evidence of learning gaps in adaptive practicing results. Trends Neurosci Educ. 2021;25: 100163. doi: 10.1016/j.tine.2021.100163 34844699 PMC8487463

[6] Spitzer MWH, Musslick S. Academic performance of K-12 students in an online-learning environment for mathematics increased during the shutdown of schools in wake of the Covid-19. PLoS One. 2021;16: 1–16. doi: 10.1371/journal.pone.0255629 34343221 PMC8330947

[7] Tomasik MJ, Helbling LA, Moser U. Educational gains of in-person vs. distance learning in primary and secondary schools: A natural experiment during the COVID-19 pandemic school closures in Switzerland. Int J Psychol. 2020;56: 566–576. doi: 10.1002/ijop.12728 33236341 PMC7753520

[8] Spitzer MWH, Gutsfeld R, Wirzberger M, Moeller K. Evaluating students ‘ engagement with an online learning environment during and after COVID-19 related school closures: A survival analysis approach. Trends Neurosci Educ. 2021;25: 100168. doi: 10.1016/j.tine.2021.100168 34844697 PMC8599139

[9] Engzell P, Frey A, Verhagen MD. Learning loss due to school closures during the COVID-19 pandemic. Proc Natl Acad Sci U S A. 2021;118. doi: 10.1073/pnas.2022376118 33827987 PMC8092566

[10] Schult J, Nicole M, Fauth B, Lindner MA. Did Students Learn Less During the COVID-19 Pandemic? Reading and Mathematics Competencies Before and After the First Pandemic Wave. 2021. https://psyarxiv.com/pqtgf/.

[11] Maldonado JE, De Witte K, Maldonado J. The effect of school closures on standardised student test outcomes. KU Leuven–Fac Econ Business. 2020; 20–48. Available: https://lirias.kuleuven.be/3189074.

[12] Gore J, Fray L, Miller A, Harris J, Taggart W. The impact of COVID-19 on student learning in New South Wales primary schools: an empirical study. Australian Educational Researcher. 2021. doi: 10.1007/s13384-021-00436-w 33727761 PMC7952260

[13] Couzin-Frankel J, Vogel G, Weiland M. Not open and shut. Science. 2020;369: 241–245. doi: 10.1126/science.369.6501.241 32675357

[14] Pereda N, Díaz-Faes DA. Family violence against children in the wake of COVID-19 pandemic: a review of current perspectives and risk factors. Child Adolesc Psychiatry Ment Health. 2020;14: 1–7. doi: 10.1186/s13034-020-00347-1 33088340 PMC7573863

[15] Cao W, Fang Z, Hou G, Han M, Xu X, Dong J, et al. The psychological impact of the COVID-19 epidemic on college students in China. Psychiatry Res. 2020;287: 1–5. Available: http://www.embase.com/search/results?subaction=viewrecord&from=export&id=L2005406993%0A 10.1016/j.psychres.2020.112934. PMC7102633 32229390

[16] Husky MM, Kovess-Masfety V, Swendsen JD. Stress and anxiety among university students in France during Covid-19 mandatory confinement. Compr Psychiatry. 2020;102: 152191. doi: 10.1016/j.comppsych.2020.152191 32688023 PMC7354849

[17] Marelli S, Castelnuovo A, Somma A, Castronovo V, Mombelli S, Bottoni D, et al. Impact of COVID-19 lockdown on sleep quality in university students and administration staff. J Neurol. 2020. doi: 10.1007/s00415-020-10056-6 32654065 PMC7353829

[18] Kovács ÁM, Téglás E, Endress AD. The Social Sense: Susceptibility to Others’ Beliefs in Human Infants and Adults. Science (80-). 2010;330: 1830–1835. doi: 10.1126/science.1190792 21205671

[19] Snape MD, Viner RM. COVID-19 in children and young people. Science (80-). 2020;370: 286–288. doi: 10.1126/science.abd6165 32958582

[20] Viner RM, Russell SJ, Croker H, Packer J, Ward J, Stansfield C, et al. School closure and management practices during coronavirus outbreaks including COVID-19: a rapid systematic review. Lancet Child Adolesc Heal. 2020;4: 397–404. doi: 10.1016/S2352-4642(20)30095-X 32272089 PMC7270629

[21] Poletti M. Hey teachers! Do not leave them kids alone! Envisioning schools during and after the coronavirus (COVID-19) pandemic. Trends Neurosci Educ. 2020;20: 100140. doi: 10.1016/j.tine.2020.100140 32917299 PMC7426210

[22] Armitage R, Nellums LB. Considering inequalities in the school closure response to COVID-19. Lancet Glob Heal. 2020;8: e644. doi: 10.1016/S2214-109X(20)30116-9 32222161 PMC7195275

[23] Vlug KFM. Because every pupil counts: The success of the pupil monitoring system in the Netherlands. Educ Inf Technol. 1997;2: 287–306. doi: 10.1023/A:1018629701040

[24] Cooper H, Nye B, Charlton K, Lindsay J, Greathouse S. The effects of summer vacation on achievement test scores: A narrative and meta-analytic review. Rev Educ Res. 1996;66: 227–268. doi: 10.3102/00346543066003227

[25] Azevedo JP, Hasan A, Goldemberg D, Aroob S, Koen Geven I. Simulating the Potential Impacts of COVID-19 School Closures on Schooling and Learning Outcomes: A Set of Global Estimates. 2020. Available: http://www.worldbank.org/prwp.%0Ahttp://pubdocs.worldbank.org/en/798061592482682799/covid-and-education-June17-r6.pdf.

[26] Kuhfeld M, Soland J, Tarasawa B, Johnson A, Ruzek E, Liu J. Projecting the Potential Impact of COVID-19 School Closures on Academic Achievement. Educ Res. 2020;49: 549–565. doi: 10.3102/0013189X20965918

[27] Atteberry A, McEachin A. School’s Out: The Role of Summers in Understanding Achievement Disparities. Am Educ Res J. 2021;58: 239–282. doi: 10.3102/0002831220937285

[28] Ironsi CS. Navigating learners towards technology-enhanced learning during post COVID-19 semesters. Trends Neurosci Educ. 2022;29: 100189. doi: 10.1016/j.tine.2022.100189 36470617 PMC9476329

[29] Heo H, Bonk CJ, Doo MY. Enhancing learning engagement during COVID-19 pandemic: Self-efficacy in time management, technology use, and online learning environments. J Comput Assist Learn. 2021;37: 1640–1652. doi: 10.1111/jcal.12603

[30] Turnbull D, Chugh R, Luck J. Transitioning to E-Learning during the COVID-19 pandemic: How have Higher Education Institutions responded to the challenge? Educ Inf Technol. 2021;26: 6401–6419. doi: 10.1007/s10639-021-10633-w 34177349 PMC8220880

[31] Nieto-Escamez FA, Roldán-Tapia MD. Gamification as Online Teaching Strategy During COVID-19: A Mini-Review. Front Psychol. 2021;12: 1–9. doi: 10.3389/fpsyg.2021.648552 34093334 PMC8175641

[32] Wang TH. Web-based dynamic assessment: Taking assessment as teaching and learning strategy for improving students’ e-Learning effectiveness. Comput Educ. 2010;54: 1157–1166. doi: 10.1016/j.compedu.2009.11.001

[33] Wang TH. Implementation of Web-based dynamic assessment in facilitating junior high school students to learn mathematics. Comput Educ. 2011;56: 1062–1071. doi: 10.1016/j.compedu.2010.09.014

[34] Marriott P. Students’ evaluation of the use of online summative assessment on an undergraduate financial accounting module. Br J Educ Technol. 2009;40: 237–254. doi: 10.1111/j.1467-8535.2008.00924.x

[35] Sun Y, Wang TH, Wang LF. Implementation of web-based dynamic assessments as sustainable educational technique for enhancing reading strategies in english class during the covid-19 pandemic. Sustain. 2021;13: 1–13. doi: 10.3390/su13115842

[36] Voelkel S. Combining the formative with the summative: The development of a twostage online test to encourage engagement and provide personal feedback in large classes. Res Learn Technol. 2013;21: 1–18. doi: 10.3402/rlt.v21i0.19153

[37] Jordan S. Student engagement with assessment and feedback: Some lessons from short-answer free-text e-assessment questions. Comput Educ. 2012;58: 818–834. doi: 10.1016/j.compedu.2011.10.007

[38] Kim NJ, Belland BR, Walker AE. Effectiveness of Computer-Based Scaffolding in the Context of Problem-Based Learning for Stem Education: Bayesian Meta-analysis. Educ Psychol Rev. 2018;30: 397–429. doi: 10.1007/s10648-017-9419-1

[39] Kim NJ, Belland BR, Lefler M, Andreasen L, Walker A, Axelrod D. Computer-Based Scaffolding Targeting Individual Versus Groups in Problem-Centered Instruction for STEM Education: Meta-analysis. Educ Psychol Rev. 2020;32: 415–461. doi: 10.1007/s10648-019-09502-3

[40] Belland BR, Walker AE, Kim NJ, Lefler M. Synthesizing Results From Empirical Research on Computer-Based Scaffolding in STEM Education: A Meta-Analysis. Rev Educ Res. 2017;87: 309–344. doi: 10.3102/0034654316670999 28344365 PMC5347356

[41] Wood D, Bruner JS, Ross G. the Role of Tutoring in Problem Solving. J Child Psychol Psychiatry. 1976;17: 89–100. doi: 10.1111/j.1469-7610.1976.tb00381.x 932126

[42] Suh S, Kim SW, Kim NJ. Effectiveness of MMORPG-based instruction in elementary English education in Korea. J Comput Assist Learn. 2010;26: 370–378. doi: 10.1111/j.1365-2729.2010.00353.x

[43] Hattie J. Visible Learning. Abingdon: Oxon: Routledge; 2008.

[44] Cepeda NJ, Vul E, Rohrer D, Wixted JT, Pashler H. Spacing effects in learning: A temporal ridgeline of optimal retention: Research article. Psychol Sci. 2008;19: 1095–1102. doi: 10.1111/j.1467-9280.2008.02209.x 19076480

[45] Cepeda N, Pashler H, Vul E, Wixted J, Rohrer D. University of California Postprints Distributed practice in verbal recall tasks: A review and quantitative synthesis A review and quantitative synthesis. Psychol Bull. 2006;132: 354–380. Available: https://cloudfront.escholarship.org/dist/prd/content/qt3rr6q10c/qt3rr6q10c.pdf. doi: 10.1037/0033-2909.132.3.354 16719566

[46] Gerbier E, Toppino TC. The effect of distributed practice: Neuroscience, cognition, and education. Trends Neurosci Educ. 2015;4: 49–59. doi: 10.1016/j.tine.2015.01.001

[47] Nazari KB, Ebersbach M. Distributed practice in mathematics: Recommendable especially for students on a medium performance level? Trends Neurosci Educ. 2019;17: 100122. doi: 10.1016/j.tine.2019.100122 31685127

[48] Toppino TC, Gerbier E. About practice. Repetition, spacing, and abstraction. 1st ed. Psychology of Learning and Motivation—Advances in Research and Theory. Elsevier Inc.; 2014. doi: 10.1016/B978-0-12-800090-8.00004–4

[49] Carpenter SK, Cepeda NJ, Rohrer D, Kang SHK, Pashler H. Using Spacing to Enhance Diverse Forms of Learning: Review of Recent Research and Implications for Instruction. Educ Psychol Rev. 2012;24: 369–378. doi: 10.1007/s10648-012-9205-z

[50] Deci EL, Ryan RM. The “what” and “why” of goal pursuits: Human needs and the self-determination of behavior. Psychol Inq. 2000;11: 227–268. doi: 10.1207/S15327965PLI1104_01

[51] Ryan RM, Deci EL. Intrinsic and extrinsic motivation from a self-determination theory perspective: Definitions, theory, practices, and future directions. Contemp Educ Psychol. 2020;61: 101860. doi: 10.1016/j.cedpsych.2020.101860

[52] Patall EA, Cooper H, Robinson JC. The Effects of Choice on Intrinsic Motivation and Related Outcomes: A Meta-Analysis of Research Findings. Psychol Bull. 2008;134: 270–300. doi: 10.1037/0033-2909.134.2.270 18298272

[53] Lewin K. Group decision and social change. Swanson GE, Newcomb TM, Hartley EL, editors. Readings in Social Psychology. New York: Holt; 1952. Available: http://www.sietmanagement.fr/wp-content/uploads/2016/04/Lewin.pdf%0Ahttp://www.sietmanagement.fr/wp-content/uploads/2016/04/Lewin.pdf.

[54] Schneider S, Nebel S, Beege M, Rey GD. The autonomy-enhancing effects of choice on cognitive load, motivation and learning with digital media. Learn Instr. 2018;58: 161–172. doi: 10.1016/j.learninstruc.2018.06.006

[55] van Loon AM, Ros A, Martens R. Motivated learning with digital learning tasks: What about autonomy and structure? Educ Technol Res Dev. 2012;60: 1015–1032. doi: 10.1007/s11423-012-9267-0

[56] Froiland JM, Oros E. Intrinsic motivation, perceived competence and classroom engagement as longitudinal predictors of adolescent reading achievement. Educ Psychol. 2014;34: 119–132. doi: 10.1080/01443410.2013.822964

[57] Vansteenkiste M, Simons J, Lens W, Soenens B, Matos L. Examining the motivational impact of intrinsic versus extrinsic goal framing and autonomy-supportive versus internally controlling communication style on early adolescents’ academic achievement. Child Dev. 2005;76: 483–501. doi: 10.1111/j.1467-8624.2005.00858.x 15784095

[58] Bates D, Mächler M, Bolker B, Walker S. Fitting Linear Mixed-Effects Models using lme4. J Stat Softw. 2014;67: 51. doi: 10.18637/jss.v067.i01

[59] R Core Team. R: A Language and Environment for Statistical Computing. Vienna, Austria; 2013. Available: http://www.r-project.org/.

[60] RStudio Team. RStudio: Integrated Development Environment for R. Bostion, MA; 2015.

[61] Spitzer MWH. Just do it! Study time increases mathematical achievement scores for grade 4–10 students in a large longitudinal cross-country study. Eur J Psychol Educ. 2022;37: 39–53. doi: 10.1007/s10212-021-00546-0 PMC8025066 40477366

[62] Spitzer MWH, Moeller K. Predicting Fraction and Algebra Achievements Online: A Large-Scale Longitudinal Study Using Data from an Online Learning Environment. Jorunal Comput Assist Learn. 2022; 1–10. doi: 10.1111/jcal.12721

[63] Siegler RS, Thompson CA, Schneider M. An integrated theory of whole number and fractions development. Cogn Psychol. 2011;62: 273–296. doi: 10.1016/j.cogpsych.2011.03.001 21569877

[64] Siegler RS, Lortie-Forgues H. Conceptual knowledge of fraction arithmetic. J Educ Psychol. 2015;107: 909–918. doi: 10.1037/edu0000025

[65] Siegler RS, Pyke AA. Developmental and individual differences in understanding of fractions. Dev Psychol. 2013;49: 1994–2004. doi: 10.1037/a0031200 23244401 PMC4103412

[66] Bailey DH, Hoard MK, Nugent L, Geary DC. Competence with fractions predicts gains in mathematics achievement. J Exp Child Psychol. 2012;113: 447–455. doi: 10.1016/j.jecp.2012.06.004 22832199 PMC3444669

[67] Booth JL, Newton KJ. Fractions: Could they really be the gatekeeper ‘ s doorman? Contemp Educ Educ Psychol. 2012;37: 247–253. doi: 10.1016/j.cedpsych.2012.07.001

[68] Ritchie SJ, Bates TC. Enduring Links From Childhood Mathematics and Reading Achievement to Adult Socioeconomic Status. Psychol Sci. 2013;24: 1301–1308. doi: 10.1177/0956797612466268 23640065

[69] Rivera-Batiz FL. Quantitative Literacy and the Likelihood of Employment among Young Adults in the United States. J Hum Resour. 1992;27: 313–328.

[70] Parsons S, Bynner J. Research report over the lifecourse. 2007. Available: https://discovery.ucl.ac.uk/id/eprint/1566242/1/Parsons2007Illuminating.pdf.

[71] Murnane RJ, Willett JB, Levy F. The Growing Importance of cognitive skills in Wage Determination. Rev Econ Stat. 1995;77: 251–266.

[72] National Mathematics Advisory Panel. Foundations for success: The final report of the National Mathematics Advisory Panel. US Dep Educ. 2008.

[73] Shenhav A, Botvinick MM, Cohen JD. The Expected Value of Control: An Integrative Theory of Anterior Cingulate Cortex Function. Neuron. 2013;79: 217–240. doi: 10.1016/j.neuron.2013.07.007 23889930 PMC3767969

[74] Musslick S, Botvinick MM, Cohen JD. A computational model of control allocation based on the Expected Value of Control. Reinforcement Learning and Decision Making Conference 2015. 2015.

[75] Brehm JW, Self EA. The intensity of motivation. Annu Rev Psychol. 1989;40: 109–131. doi: 10.1146/annurev.ps.40.020189.000545 2648973

[76] Cerasoli CP, Nicklin JM, Ford MT. Intrinsic motivation and extrinsic incentives jointly predict performance: A 40-year meta-analysis. Psychol Bull. 2014;140: 980–1008. doi: 10.1037/a0035661 24491020

[77] Stella M. Network psychometrics and cognitive network science open new ways for understanding math anxiety as a complex system. J Complex Networks. 2022;10: 5–8. doi: 10.1093/comnet/cnac022

[78] Devine A, Hill F, Carey E, Szűcs D, Devine A. Cognitive and emotional math problems largely dissociate: Prevalence of developmental dyscalculia and mathematics anxiety. J Educ Psychol. 2016;110: 431–444. Available: https://www.airipa.it/wp-content/uploads/2019/03/devine_etal2018.pdf.

[79] Ashcraft MH, Kirk EP. The relationships among working memory, math anxiety, and performance. J Exp Psychol Gen. 2001;130: 224–237. doi: 10.1037//0096-3445.130.2.224 11409101

[80] Ashcraft M, Krause J. Working memory, math performance, and math anxiety. Psychon Bull Rev. 2007;14: 243–248. Available: http://personal.psu.edu/users/s/a/sap246/spaul_HCOMPworkshop_CHI11.pdf%5Cnpapers3://publication/uuid/32ECD18C-4D33-42EB-802D-D2EAEA9FEA2E. doi: 10.3758/bf03194059 17694908

[81] Carey E, Devine A, Hill F, Szucs D. Differentiating anxiety forms and their role in academic performance from primary to secondary school. PLoS One. 2017;12: 1–20. doi: 10.1371/journal.pone.0174418 28350857 PMC5370099

[82] Caviola S, Toffalini E, Giofrè D, Ruiz JM, Szűcs D, Mammarella IC. Math Performance and Academic Anxiety Forms, from Sociodemographic to Cognitive Aspects: a Meta-analysis on 906,311 Participants. Educational Psychology Review. 2022. doi: 10.1007/s10648-021-09618-5

[83] Jez SJ, Wassmer RW. The Impact of Learning Time on Academic Achievement. Educ Urban Soc. 2015;47: 284–306. doi: 10.1177/0013124513495275

[84] Roschelle J, Murphy RF, Mason CA. Online Mathematics Homework Increases Student Achievement. 2016;2. doi: 10.1177/2332858416673968

[85] Zimmerman BJ. Self-Regulated Learning and Academic Achievement: An Overview. Educ Psychol. 1990;25: 3–17. doi: 10.1207/s15326985ep2501_2

[86] Pelikan ER, Lüftenegger M, Holzer J, Korlat S, Spiel C, Schober B. Learning during COVID-19: the role of self-regulated learning, motivation, and procrastination for perceived competence. Zeitschrift fur Erziehungswiss. 2021;24: 393–418. doi: 10.1007/s11618-021-01002-x 33686344 PMC7931168

[87] Blume F, Schmidt A, Kramer AC, Schmiedek F, Neubauer AB. Homeschooling during the SARS-CoV-2 pandemic: the role of students’ trait self-regulation and task attributes of daily learning tasks for students’ daily self-regulation. Zeitschrift fur Erziehungswiss. 2021;24: 367–391. doi: 10.1007/s11618-021-01011-w 33821144 PMC8014902

[88] Chiu TKF. Applying the self-determination theory (SDT) to explain student engagement in online learning during the COVID-19 pandemic. J Res Technol Educ. 2022;54: S14–S30. doi: 10.1080/15391523.2021.1891998

[89] Baker R, Roll I, Corbett A, Koedinger KR, Walonoski J, Heffernan N. Why students engage in “gaming the system” behavior in interactive learning environments. J Interact Learn Res. 2008;19: 185–224. Available: http://www.columbia.edu/~rsb2162/JILR1902Baker2.pdf.

