# Supplementary material for: Assignment strategies modulate students’ academic performance in an online learning environment during the first and second COVID-19 related school closures
Source: PLoS One. 2023 May 3;18(5):e0284868. doi: 10.1371/journal.pone.0284868 (PMC10155976; doi:10.1371/journal.pone.0284868)
Supplement: S1 Text — (DOCX) [file pone.0284868.s001.docx]

**Supplementary Material**

**Material**

We analyzed performance across three major mathematical topics (i.e., fractions, percentages, linear equations) covered in a total of eight books. Below, we describe each of the eight books and depict examples thereof in Figure 1.

***Basics of fractions***

This book comprises four different chapters. The first chapter “Representing Fractions” includes 11 different problem sets on how fractions can be represented (e.g., $decomposing the fraction\frac{3}{8}into sums of unit fractions with the same denominator; answer:\frac{1}{8}+\frac{1}{8}+\frac{1}{8}$). The second chapter “Simplifying and Expanding Fractions” includes 13 different problem sets (e.g., $finding an expanded fraction of\frac{17}{18}; answer:\frac{134}{144}$). The third chapter includes 10 problem sets on “Parts of a Quantity and Percentages” (e.g., $converting \frac{1}{5}m into cm; answer: \frac{1}{5}of 100 cm$). The fourth and final chapter “Improper Fractions and Mixed Numbers” includes 7 different problem sets (e.g., on identifying proper fractions out of a set of numbers; see Panel 1 in Figure 1 for an example).

***Addition and subtraction of fractions***

This book comprises three different chapters. The first chapter “Addition and Subtraction of Fractions with Common Denominators” includes eight different problem sets (e.g., $calculate the following:\frac{9}{25}+\frac{3}{25}= ?; answer:\frac{12}{25}$). The second chapter “Addition of Fractions with Different Denominators” includes 10 different problem sets (e.g., $\frac{3}{11}+\frac{9}{22}= ?; answer:\frac{15}{22}$). The third “Subtraction of Fractions with Different Denominators” includes nine different problem sets (e.g., $\frac{4}{5}-\frac{2}{9}= ?; answer: \frac{26}{45}$).

***Multiplication and division of fractions***

This book comprises three different chapters. The first chapter “Multiplication of Fractions” includes 11 different problem sets (e.g., $calculate the following:\frac{4}{9}*\frac{2}{5}= ?; answer: 0.178$). The second chapter “Division of Fractions” includes 10 different problem sets (e.g., $calculate:\frac{5}{7}\div\frac{8}{9}= ?; answer: 0.803$). The third chapter “Calculating with Several Fractions” includes seven different problem sets (e.g., $\frac{16}{45}\div\frac{6}{25}\times\frac{7}{8}= ?; answer: 1.3$).

***Calculating percents***

This book comprises five different chapters. The first chapter “Representing Percents” includes 10 different problem sets (e.g., $convert the fraction to percent: \frac{77}{100} = ?; answer: 77\%$). The second chapter “Calculations on Percent” includes eight different problem sets (e.g., $calculate: 20\% of 203= ?; answer: 40.6$). The third chapter “Calculations on Percent Using the Rule of Three” includes five different problem sets applying the rule of three. The fourth chapter “Calculation on Percent Using Formulas” includes three text problem sets on which students must apply percentages in formulars (see Panel 2 in Figure 1). The fifth chapter “Percentage Increase and Decrease” includes eight text-based problem sets on which students must calculate for example the increase of an advertisement cost if a manager decides to increase his investment by 23% (see Panel 3 in Figure 1).

***Calculating interest***

This book comprises two different chapters. The first chapter “Calculate with annual interest” includes six different problem sets (e.g., y$ou earn interest of 7510\$ at a rate of 7.1\% -calculate the interest; answer: 533,21\$$). The second chapter “Calculating Interest for Periods Under One Year and Compound Interest” includes four different problem sets (e.g., calculating the compound interest).

***Linear equations***

This book comprises three different chapters. The first chapter “Recognizing and Setting up Linear Equations” includes nine different problem sets (see Panel 4 in Figure 1 for an example). The second chapter “Solving Equations by Rearranging” includes 13 different problem sets (e.g., $use the balance method to solve the linear equation: 2p + 21 = 35; answer: p = 7$). The third chapter “Applications on Equations” includes six different problem sets (e.g., $Mike is 3 times older than Ethan. The age difference between the two is 18 years. How old is Ethan?; answer: 9 (3x-x=18)$).

***Linear inequalities***

This book comprises three different chapters. The first chapter “Inequalities Key Terms” includes four different problem sets (e.g., $on identifying and setting up linear inequalities represented on a balance scale$). The second chapter “Solving Inequalities” includes eight different problem sets (e.g., $what value of x satisfy the linear inequality 4x - 5 < 15; answer: x < 5$). The third chapter “Applying Inequalities” includes four different problem sets (e.g., $create inequalities from real-world situations and solve them step by step$).

***Systems of linear equations***

This book comprises five different chapters. The first chapter “Linear Equations in two Variables” includes four different problem sets (e.g., $finding solutions of linear equations in two variables; 6x + 3y + 3 = 0; answer: (-5, -11)$). The second chapter “Solving Systems of Linear Equations Graphically” includes five different problem sets on finding the solution of two linear equations involving unknowns x and y. The third chapter “Solving Systems of Linear Equations using Substitution and the Equating Method” includes seven different problem sets on this topic. The fourth chapter “Solving Systems of Linear Equations by Elimination” includes six problem sets. The fifth chapter “Applications on Systems of Linear Equations” includes 10 text-based problem sets on which students must apply systems of linear equations.

**Results**

**Fractions**

Results from the fraction analyses are depicted in Figures 3 and 4. In the following sections, we describe each individual analysis.


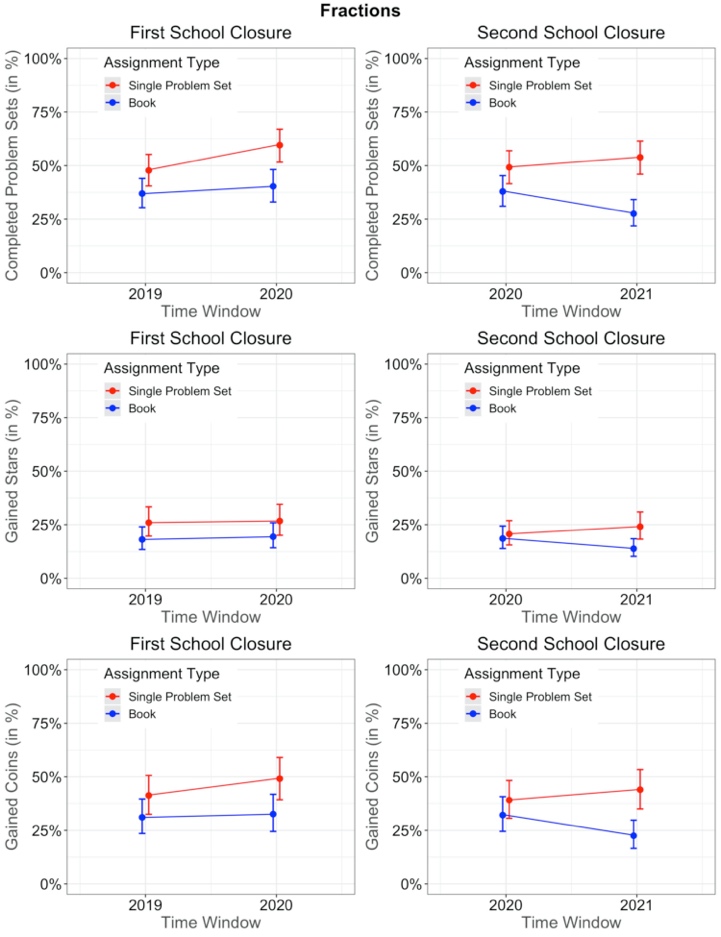


**Figure 3. Estimates for performance on fraction books for single problem set assignments (red) and book assignments (blue), for the school closure in 2020 (left panel) and 2021 (right panel) compared to the same time periods in the preceding years.** Vertical bars indicate the standard error of the mean. Students showed greater completion rates, gained more stars, and gained more coins when single problem sets were assigned, compared to entire book assignments. In addition, students completed more problem sets and gained more coins during the first and second school closure when they were assigned single problem sets by their teachers, compared to the same time period in the previous year. This pattern does not replicate in cases where teachers assigned entire book topics. Finally, students gained more stars during the second lockdown as compared to the same time period in the previous year in cases where teachers assigned single problem sets but not entire books.


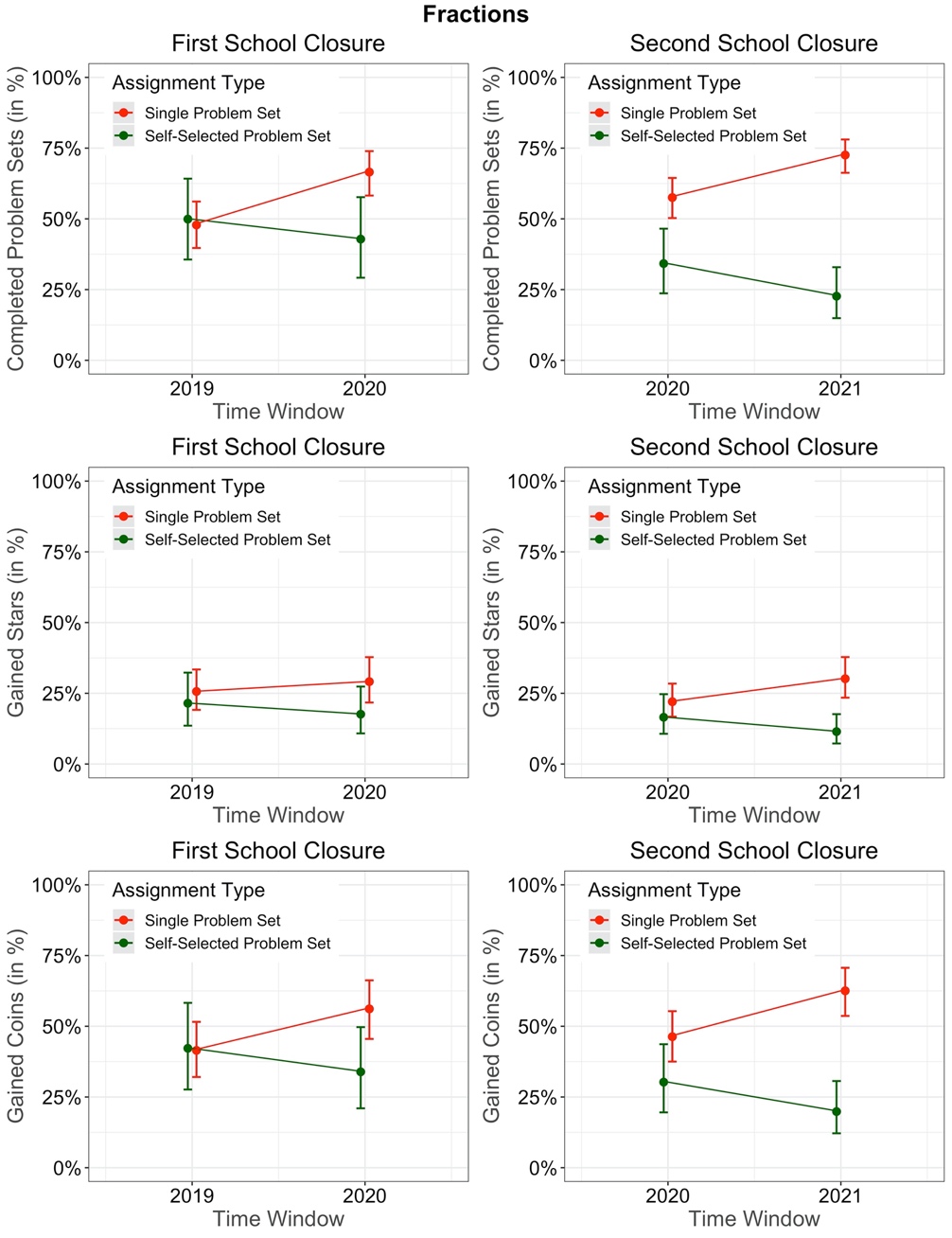


**Figure 4. Estimates for performance on fraction books for single problem set assignments (red) and self-selected problem sets (green), for the school closure in 2020 (left panel) and 2021 (right panel) compared to the same time periods in the preceding years.** Vertical bars indicate the standard error of the mean. All plots indicate a similar pattern of results: students completed more problem sets, gained more stars, and gained more coins during the first and second school closure if they were assigned single problem sets as compared to the same time periods in the preceding years. We did not observe this pattern for cases in which students self-selected problem sets.

***First shutdown of schools***

**Completion Rate (single problem sets vs. entire books).** The main effect of time window was significant (*b* = 0.15; *z* = 3.04; *p* = .002), with more completed problem sets during school closure as compared to the same time window in the previous year. The main effect of assignment was significant (*b* = 0.31; *z*= 10.93; *p* < .001), with higher completion rates when individual problem sets were assigned compared to problem sets assigned as entire books. The interaction of time window and assignment was significant (*b* = .08; *z* = 2.98; *p* = .003), suggesting that the increase in completion rate associated with the lockdown was larger for students who got assigned single problem sets as compared to entire books.

**Completion Rate (single problem sets vs. self-selected problem sets)**. The main effect of time window was significant (*b* = 0.12; *z* = 3.09; *p* = .001), with overall more completed problem sets during school closure as compared to the same time window in the previous year. The main effect of assignment was not significant (*b* = 0.22; *z* = 0.88; *p* = .380). The interaction of time window and assignment was significant (*b* = .26; *z* = 6.70; *p* < .001), indicating that that the increase in completion rate associated with the lockdown was larger for students who got assigned single problem sets by their teachers relative to students who selected their own problem sets.

**Stars (single problem sets vs. entire books).** The main effect of time window was not significant (*b* = .03; *z* = 0.68; *p* = .496). The main effect of assignment was significant (*b* = .22; *z* = 7.85; *p* < .001), with more stars gained on single problem set assignments compared to entire book assignments. The interaction of time window and assignment was not significant (*b* = -.01; *z* = -0.40; *p* = .690).

**Stars *(*single problem sets vs. self-selected problem sets).** The main effect of time window was not significant (*b* = -.02; *z* = -0.46; *p* = .643). The main effect of assignment was not significant (*b* = .22; *z* = 1.03; *p* = .304). The interaction of time window and assignment was significant (*b* = .10; *z* = 2.75; *p* = .006), indicating that students who got assigned single problem sets by their teachers showed a greater increase in stars during the lockdown relative to students who self-selected their problem sets.

**Coins (single problem sets vs. entire books).** The main effect of time window was not significant (*b* = .10; *z* = 1.87; *p* = .061). The main effect of assignment was significant (*b* = .28; *z* = 10.12; *p* < .001), with more coins gained on single problem set assignments compared to problem sets assigned via an entire book assignment. The interaction of time window and assignment was significant (*b* = .06; *z* = 2.22; *p* = .027) suggesting that students who got assigned single problem sets showed a greater increase in coins during the lockdown relative to students who got assigned entire books.

**Coins (single problem sets vs. self-selected problem sets).** The main effect of time window was not significant (*b* = .06; *z* = 1.46; *p* = .144). The main effect of assignment was not significant (*b* = .22; *z* = 0.84; *p* = .401). The interaction of time window and assignment was significant (*b* = .24; *z* = 5.83; *p* < .001) indicating that students who got assigned single problem sets by their teachers showed a greater increase in coins during the lockdown relative to students who self-selected their problem sets.

***Second lockdown***

**Completion Rate (single problem sets vs. entire books).** The main effect of time window was not significant (*b* = -.07; *z* = -1.31; *p* = .190). The main effect of assignment was significant (*b* = .39; *z*= 10.16; *p* < .001), with higher completion rates on problem sets when they were assigned one by one as opposed to as entire books. The interaction of time window and assignment was significant (*b* = .16; *z* = 4.33; *p* < .001), suggesting that the increase in completion rate associated with single problem sets was larger for the lockdown period compared to the same time frame in the previous year.

**Completion Rate (single problem sets vs. self-selected problem sets)**. The main effect of time window was not significant (*b* = .02; *z* = 0.607; *p* = .543). The main effect of assignment was significant (*b* = .79; *z* = 3.53; *p* < .001), suggesting that students complete more problem sets if they were assigned by the teachers as opposed to self-selected. The interaction of time window and assignment was significant (*b* = .31; *z* = 7.88; *p* < .001), indicating that the increase in completion rate associated with single problem sets was larger for the lockdown period compared to the same time frame in the previous year.

**Stars (single problem sets vs. entire books).** The main effect of time window was not significant (*b* = -.03; *z* = -0.80; *p* = .422). The main effect of assignment was significant (*b* = .20; *z* = 5.37; *p* < .001), with more stars gained on single problem set assignments, compared to entire books assignments. The interaction of time window and assignment was significant (*b* = .14; *z* = 3.70; *p* < .001), indicating that the difference between the two assignment types with respect to stars gained increased during the second school closure as compared to the same time window the year before.

**Stars *(*single problem sets vs. self-selected problem sets).** The main effect of time window was not significant (*b* < -.01; *z* = -0.01; *p* = .990). The main effect of assignment was significant (*b* = .39; *z* = 1.99; *p* = .046), with more stars gained on single problem sets were assigned, compared to self-selected problem sets. The interaction of time window and assignment was significant (*b* = .21; *z* = 5.53; *p* < .001), indicating that the increase in gained starts associated with single problem sets was larger for the lockdown period compared to the same time frame in the previous year.

**Coins (single problem sets vs. entire books).** The main effect of time window was not significant (*b* = -.07; *z* = -1.28; *p* = .200). The main effect of assignment was significant (*b* = .32; *z* = 8.36; *p* < .001), with more coins gained on single problem set assignments compared to book assignments. The interaction of time window and assignment was significant (*b* = .17; *z* = 4.54; *p* < .001) with the difference in coins gained between the two assignment types increasing during school closures compared to the same time period in the previous year.

**Coins (single problem sets vs. self-selected problem sets).** The main effect of time window was not significant (*b* = .02; *z* = 0.61; *p* = .537). The main effect of assignment was significant (*b* = .65; *z* = 2.72; *p* = .006), with more coins gained on single problem set assignments, compared to self-selected problem sets. The interaction of time window and assignment was significant (*b* = .31; *z* = 7.64; *p* < .001) with the difference on coins gained between the two assignment types increasing during the lockdown compared to the same time period in the previous year.

**Percentages & Interest**

Results from the percentages and interest analyses are shown in Figure 5 and Figure 6. We report the results of these analyses in the following sections.

**
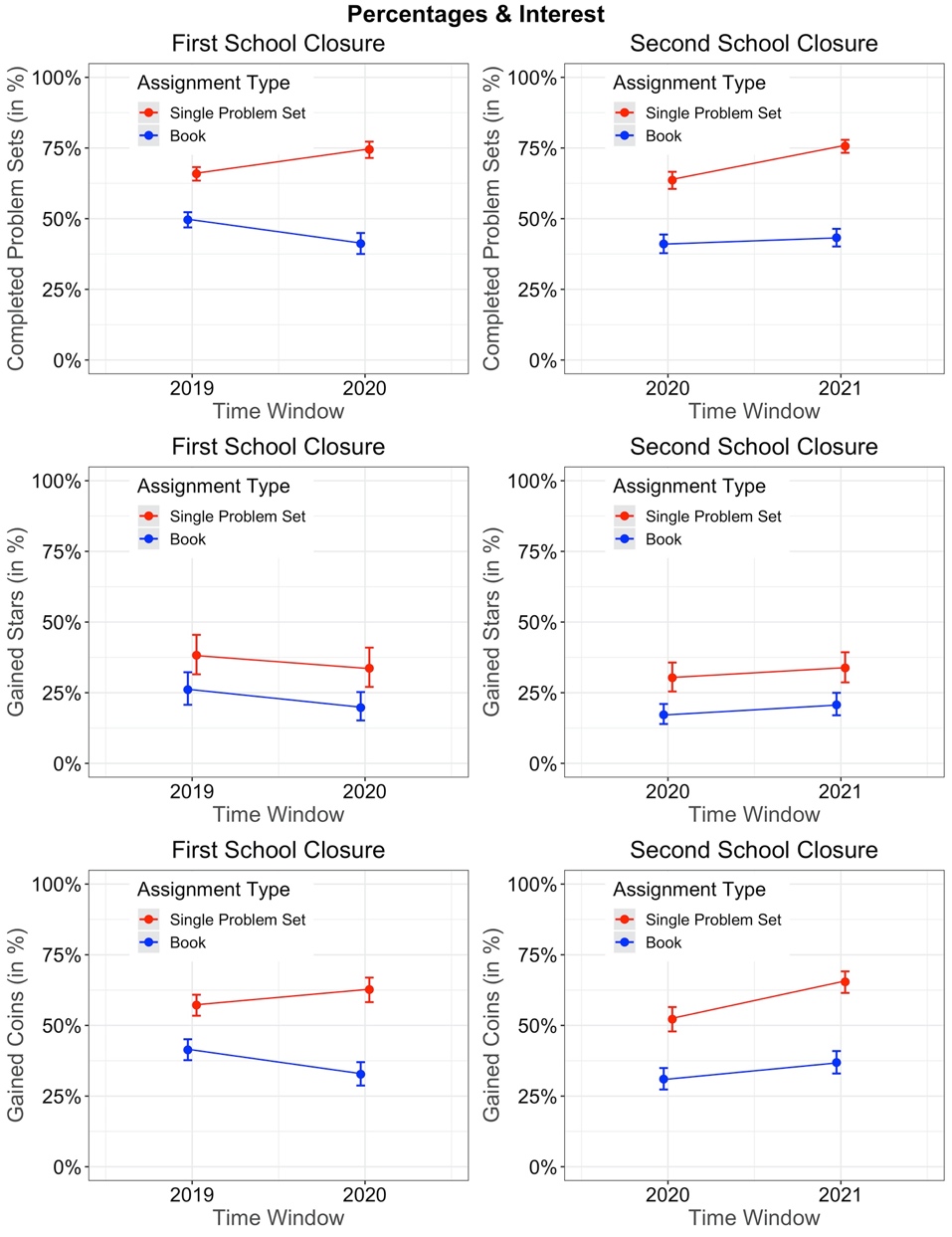
**

**Figure 5. Estimates for performance on percentages and interest books for single problem set assignments (red) and book assignments (blue), for the school closure in 2020 (left panel) and 2021 (right panel) compared to the same time periods in the preceding years.** Vertical bars indicate the standard error of the mean. Students overall completed more problem sets, gained more stars, and collected more coins when they got assigned single problem sets as opposed to entire books. In addition, students who got assigned single problem sets completed more problems and collected more coins during both school closures, relative to the same time periods in the previous years. Students who got assigned entire books did not show such a consistent pattern.

**
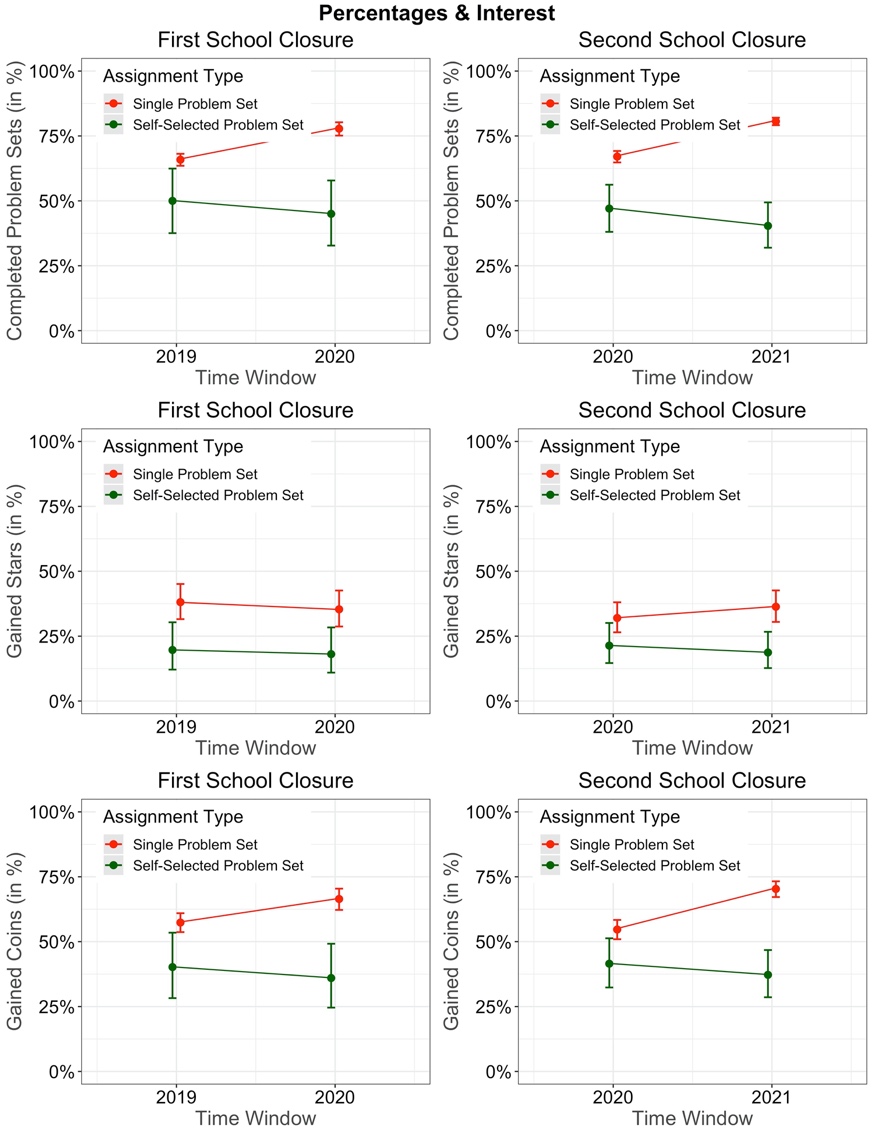
**

**Figure 6. Estimates for performance on percentages and interest books for single problem set assignments (red) and self-selected problem sets (green), for the school closure in 2020 (left panel) and 2021 (right panel) compared to the same time periods in the preceding years.** Vertical bars indicate the standard error of the mean. Students completed more problems, gained more stars, and collected more coins when they completed single problem sets assigned by their teachers relative to when they performed problem selected by themselves. In addition, students who got assigned single problem sets completed more problems and collected more coins during both school closures, relative to the same time periods in the previous years. Students who selected their own problem sets did not show such a consistent pattern.

***First lockdown***

**Completion Rate (single problem sets vs. entire books).** The main effect of time window was not significant (*b* = .01; *z* = 0.33; *p* = .737). The main effect of assignment was significant (*b* = .52; *z*= 18.06; *p* < .001), indicating higher completion rates for single problem set assignments. The interaction of time window and assignment was significant (*b* = .18; *z* = 6.48; *p* < .001), indicating that the increase in problem set completions associated with single problem sets was larger for the lockdown period compared to the same time frame in the previous year.

**Completion Rate (single problem sets vs. self-selected problem sets)**. The main effect of time window was significant (*b* = .10; *z* = 2.66; *p* = .007), with overall more completed problem sets during school closure as compared to the same time window in the previous year. The main effect of assignment was significant (*b* = .52; *z* = 2.04; *p* < .001) with higher completion rates when individual problem sets were assigned compared to when students self-selected problem sets. The interaction of time window and assignment was significant (*b* = .20; *z* = 5.36; *p* < .001), suggesting that the increase in completion rate associated with the lockdown was larger for students who got assigned single problem sets by their teachers as opposed to students who selected problem sets themselves.

**Stars (single problem sets vs. entire books).** The main effect of time window was significant (*b* = -.14; *z* = -2.87; *p* = .004), with more stars gained during school closure as compared to the same time window one year before. The main effect of assignment was significant (*b* = .32; *z* = 10.90; *p* < .001), with more stars gained on single problem set assignments, compared to entire book assignments. The interaction of time window and assignment was not significant (*b* = .04; *z* = 1.36; *p* = .172).

**Stars *(*single problem sets vs. self-selected problem sets).** The main effects of time window (*b* = -.05; *z* = -1.47; *p* = .141) and assignment (*b* = .45; *z* = 1.77; *p* = .077), as well as their interaction (*b* = -.003; *z* = -0.92; *p* = .926) were not significant.

**Coins (single problem sets vs. entire books).** The main effect of time window was not significant (*b* = -.035; *z* = -0.66; *p* = .507). The main effect of assignment was significant (*b* = .46; *z* = 16.46; *p* < .001), with more coins gained on single problem set assignments compared to problem sets assigned via a entire book assignment. The interaction of time window and assignment was significant (*b* = .14; *z* = 5.26; *p* < .001) with the difference on coins gained between the two assignment types increasing during school closures compared to the year before.

**Coins (single problem sets vs. self-selected problem sets).** The main effect of time window was not significant (*b* = .05; *z* = 1.40; *p* = .162). The main effect of assignment was not significant (*b* = .49; *z* = 1.82; *p* = .068). The interaction of time window and assignment was significant (*b* = .14; *z* = 3.82; *p* < .001) with the difference in coins gained between the two assignment types increasing during school closures compared to the same time period in the previous year.

***Second lockdown***

**Completion Rate (single problem sets vs. entire books).** The main effect of time window was significant (*b* = 0.17; *z* = 2.96; *p* = .003) with higher completion rates during the lockdown as compared to before. The main effect of assignment was significant (*b* = 0.58; *z*= 15.58; *p* < .001), with higher completion rates on problem sets when single problem sets were assigned compared to entire book assignments. The interaction of time window and assignment was significant (*b* = .12; *z* = 3.25; *p* = .001), suggesting that the increase in completion rate associated with the lockdown was larger for students who got assigned single problem sets as compared to entire books.

**Completion Rate (single problem sets vs. self-selected problem sets)**. The main effect of time window was significant (*b* = .11; *z* = 2.84; *p* = .004) with overall more completed problem sets during school closure as compared to the same time window in the previous year. The main effect of assignment was significant (*b* = .66; *z* = 3.52; *p* < .001) with higher completion rates when individual problem sets were assigned compared to when students self-selected problem sets. The interaction of time window and assignment was significant (*b* = .24; *z* = 6.31; *p* < .001), suggesting that the increase in completion rate associated with the lockdown was larger for students who got assigned single problem sets by their teachers as compared to students who selected problem sets themselves.

**Stars (single problem sets vs. entire books).** The main effect of time window was not significant (*b* = .09; *z* = 1.72; *p* = .08). The main effect of assignment was significant (*b* = .35; *z* = 9.03; *p* < .001), with more stars gained on single problem set assignments, compared to entire book assignments. The interaction of time window and assignment was not significant (*b* = -.02; *z* = -0.45; *p* = .651).

**Stars (single problem sets vs. self-selected problem sets).** The main effect of time window was not significant (*b* = .01; *z* = 0.17; *p* = .866). The main effect of assignment was not significant (*b* = .36; *z* = 1.83; *p* = .066) with more stars gained when single problem sets were assigned, compared to self-selected problem sets. The interaction of time window and assignment was significant (*b* = .089; *z* = 2.18; *p* = .029), indicating that the increase in gained starts associated with single problem sets was larger for the lockdown period compared to the same time frame in the previous year.

**Coins (single problem sets vs. entire books).** The main effect of time window was significant (*b* = .20; *z* = 3.74; *p* < .001), with more coins gained during school closures as compared to the same time window in the previous year. The main effect of assignment was significant (*b* = .52; *z* = 14.01; *p* < .001), with more coins gained on single problem set assignments compared to book assignments. The interaction of time window and assignment was significant (*b* = .07; *z* = 1.95; *p* = .001) suggesting that the increase in collected coins associated with the lockdown was larger for students who got assigned single problem sets as compared to entire books.

**Coins (single problem sets vs. self-selected problem sets).** The main effect of time window was not significant (*b* = .12; *z* = 3.20; *p* = .001) with more coins gained during school closures as compared to the same time window in the previous year. The main effect of assignment was significant (*b* = .48; *z* = 2.44; *p* = .013) with more coins gained on single problem set assignments, compared to self-selected problem sets. The interaction of time window and assignment was significant (*b* = .21; *z* = 5.53; *p* < .001) indicating that the increase in collected coins associated with single problem sets was larger for the lockdown period compared to the same time frame in the previous year.

**Linear equations**

Linear equations results are depicted in Figure 7 and Figure 8. The following sections describe the results of each analysis in detail.

**
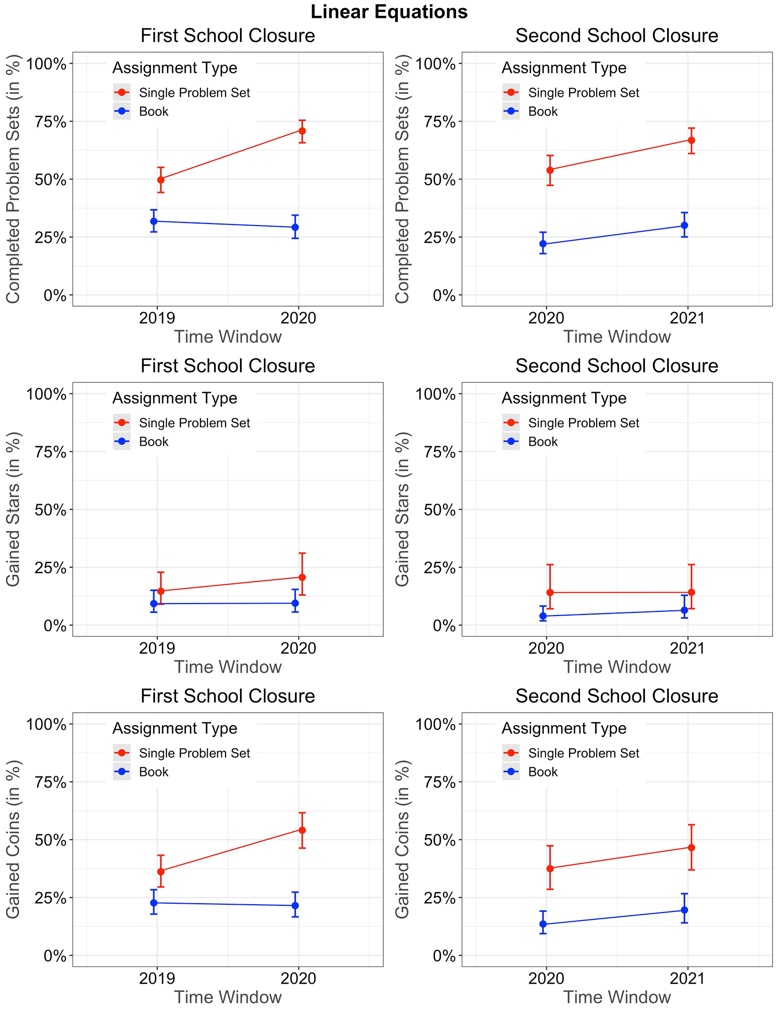
**

**Figure 7. Estimates for performance on linear equation books for single problem set assignments (red) and book assignments (blue) and for the first (left panel), for the school closure in 2020 (left panel) and 2021 (right panel) compared to the same time periods in the preceding years.** Vertical bars indicate the standard error of the mean. Students completed more problems, gained more stars, and collected more coins when they got assigned single problem as compared to entire books in the first and second period of school closures. In addition, students who got assigned single problem sets completed more problems, gained more stars, and collected more coins during the first school closure in 2020, relative to the same time period in the previous years. Students who got assigned entire books did not show such a consistent pattern.


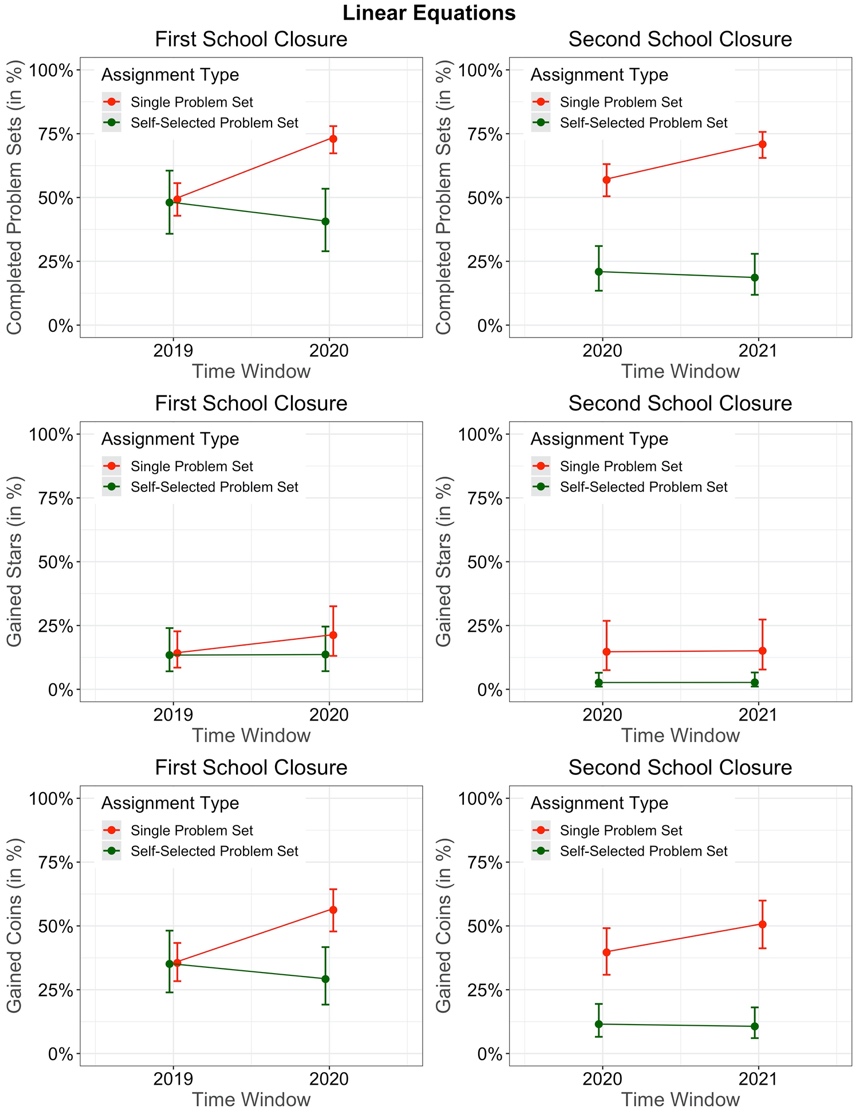


**Figure 8. Estimates for performance on linear equation books for single problem set assignments (red) and self-selected problem sets (green), for the school closure in 2020 (left panel) and 2021 (right panel) compared to the same time periods in the preceding years.** Vertical bars indicate the standard error of the mean. Students completed more problems, gained more stars, and collected more coins when they got assigned single problem by their teachers as compared to when they selected problem sets themselves. In addition, students who got assigned single problem sets completed more problems, gained more stars, and collected more coins during the first school closure in 2020, relative to the same time period in the previous years. Students who selected problem sets themselves did not show such a consistent pattern.

***First lockdown***

**Completion Rate (single problem sets vs. entire books).** The main effect of time window was significant (*b* = .19; *z* = 3.70; *p* < .001), with more problem sets completed during school closures as compared to the same time window one year before. The main effect of assignment was significant (*b* = .63; *z*= 18.32; *p* < .001), with higher completion rates when individual problem sets were assigned compared to problem sets assigned via a entire book assignment. The interaction of time window and assignment was significant (*b* = .26; *z* = 7.43; *p* < .001), indicating that the increase in completion rate associated with the lockdown was larger for students who got assigned single problem sets as compared to entire books.

**Completion Rate (single problem sets vs. self-selected problem sets)**. The main effect of time window was significant (*b* = .18; *z* = 4.45; *p* < .001), with overall more completed problem sets during school closure as compared to the same time window in the previous year. The main effect of assignment was not significant (*b* = .35; *z* = 1.56; *p* = .118). The interaction of time window and assignment was significant (*b* = .33; *z* = 8.17; *p* < .001), indicating that the increase in completed problem sets associated with the lockdown was larger for students who got assigned problem sets by their teachers as opposed to students who selected problem sets themselves.

**Stars (single problem sets vs. entire books).** The main effect of time window was significant (*b* = .11; *z* = 2.13; *p* = .033), with more stars gained during school closure as compared to the same time window one year before. The main effect of assignment was significant (*b* = .35; *z* = 9.50; *p* < .001), with more stars gained on single problem set assignments, compared to entire book assignments. The interaction of time window and assignment was significant (*b* = .09; *z* = 2.63; *p* = .008), suggesting that the increase in accumulated stars associated with the lockdown was larger for students who got assigned single problem sets as compared to entire books.

**Stars *(*single problem sets vs. self-selected problem sets).** The main effect of time window was significant (*b* = .12; *z* = 2.74; *p* = .141) , with more stars gained during school closure as compared to the same time window one year before. The main effect of assignment was not significant (*b* = .14; *z* = 0.69; *p* = .492). The interaction of time window and assignment was significant (*b* = .12; *z* = 2.52; *p* = .011) , indicating that the increase in accumulated stars associated with the lockdown was larger for students who got assigned problem sets by their teachers as opposed to students who selected problem sets themselves.

**Coins (single problem sets vs. entire books).** The main effect of time window was significant (*b* = -16; *z* = 3.19; *p* = .001), with more coins gained during school closure as compared to before. The main effect of assignment was significant (*b* = .53; *z* = 15.32; *p* < .001), with more coins gained on single problem set assignments compared to problem sets assigned via a entire book assignment. The interaction of time window and assignment was significant (*b* = .20; *z* = 5.81; *p* < .001) suggesting that the increase in accumulated coins associated with the lockdown was larger for students who got assigned single problem sets as compared to entire books.

**Coins (single problem sets vs. self-selected problem sets).** The main effect of time window was significant (*b* = .14; *z* = 3.52; *p* < .001), with more coins gained during school closure as compared to before. The main effect of assignment was not significant (*b* = .29; *z* = 1.29; *p* = .196). The interaction of time window and assignment was significant (*b* = .28; *z* = 6.83; *p* < .001) suggesting that the increase in accumulated coins associated with the lockdown was larger for students who got assigned problem sets by their teachers as opposed to students who selected problem sets themselves.

***Second lockdown***

**Completion Rate (single problem sets vs. entire books).** The main effect of time window was significant (*b* = 0.24; *z* = 3.56; *p* < .001) with higher completion rates during the lockdown as compared to before. The main effect of assignment was significant (*b* = 0.74; *z*= 12.40; *p* < .001), with higher completion rates on problem sets when single problem sets were assigned compared to entire book assignments. The interaction of time window and assignment was not significant (*b* = .04; *z* = 0.55; *p* = .582).

**Completion Rate (single problem sets vs. self-selected problem sets)**. The main effect of time window was significant (*b* = .11; *z* = 2.60; *p* = .009), with overall more completed problem sets during school closure as compared to the same time window in the previous year. The main effect of assignment was significant (*b* = .99 *z* = 4.08; *p* < .001) with higher completion rates when individual problem sets were assigned compared to when students self-selected problem sets. The interaction of time window and assignment was significant (*b* = .19; *z* = 4.24; *p* < .001), suggesting that the increase in completion rate associated with the lockdown was larger for students who got assigned single problem sets by teachers as compared students who selected problem sets themselves.

**Stars (single problem sets vs. entire books).** The main effect of time window was not significant (*b* = .13; *z* = 1.61; *p* = .10). The main effect of assignment was significant (*b* = .56; *z* = 7.58; *p* < .001), with more stars gained on single problem set assignments, compared to entire book assignments. The interaction of time window and assignment was not significant (*b* = -.12; *z* = -1.72; *p* = .085).

**Stars *(*single problem sets vs. self-selected problem sets).** The main effect of time window was not significant (*b* = .01; *z* = 0.22; *p* = .828). The main effect of assignment was significant (*b* = .92; *z* = 3.22; *p* = .001), with more stars gained on single problem set assignments, compared to entire book assignments. The interaction of time window and assignment was not significant (*b* = .01; *z* = 0.07; *p* = .944).

**Coins (single problem sets vs. entire books).** The main effect of time window was significant (*b* = .20; *z* = 2.93; *p* = .003), with more coins gained during school closures as compared to the same time window in the previous year. The main effect of assignment was significant (*b* = .65; *z* = 10.49; *p* < .001), with more coins gained on single problem set assignments compared to book assignments. The interaction of time window and assignment was not significant (*b* = -.01; *z* = -0.28; *p* = .778).

**Coins (single problem sets vs. self-selected problem sets).** The main effect of time window was significant (*b* = .09; *z* = 1.95; *p* = .050), with more coins gained during school closures as compared to the same time window in the previous year. The main effect of assignment was significant (*b* = .94; *z* = 3.72; *p* < .001), with more coins gained on single problem set assignments compared to book assignments. The interaction of time window and assignment was significant (*b* = .13; *z* = 2.92; *p* = .003) suggesting that the increase in accumulated coins associated with the lockdown was larger for students who got assigned single problem sets by teachers as compared to students who selected problem sets themselves.
